# Translatomics combined with transcriptomics and proteomics reveals novel functional, recently evolved orphan genes in *Escherichia coli* O157:H7 (EHEC)

**DOI:** 10.1186/s12864-016-2456-1

**Published:** 2016-02-24

**Authors:** Klaus Neuhaus, Richard Landstorfer, Lea Fellner, Svenja Simon, Andrea Schafferhans, Tatyana Goldberg, Harald Marx, Olga N. Ozoline, Burkhard Rost, Bernhard Kuster, Daniel A. Keim, Siegfried Scherer

**Affiliations:** Lehrstuhl für Mikrobielle Ökologie, Zentralinstitut für Ernährungs- und Lebensmittelforschung, Wissenschaftszentrum Weihenstephan, Technische Universität München, Weihenstephaner Berg 3, 85354 Freising, Germany; Lehrstuhl für Datenanalyse und Visualisierung, Fachbereich Informatik und Informationswissenschaft, Universität Konstanz, Box 78, 78457 Konstanz, Germany; Department of Informatics - Bioinformatics & TUM-IAS, Technische Universität München, Boltzmannstraße 3, 85748 Garching, Germany; Chair of Proteomics and Bioanalytics, Wissenschaftszentrum Weihenstephan, Technische Universität München, Emil-Erlenmeyer-Forum 5, 85354 Freising, Germany; Institute of Cell Biophysics, Russian Academy of Sciences, Moscow Region, 142290 Pushchino, Russia; Bavarian Center for Biomolecular Mass Spectrometry (BayBioMS), Technische Universität München, Gregor-Mendel-Str. 4, 85354 Freising, Germany

**Keywords:** Translatome, Riboseq, Proteome, Novel genes, Orphans, EHEC

## Abstract

**Background:**

Genomes of *E. coli*, including that of the human pathogen *Escherichia coli* O157:H7 (EHEC) EDL933, still harbor undetected protein-coding genes which, apparently, have escaped annotation due to their small size and non-essential function. To find such genes, global gene expression of EHEC EDL933 was examined, using strand-specific RNAseq (transcriptome), ribosomal footprinting (translatome) and mass spectrometry (proteome).

**Results:**

Using the above methods, 72 short, non-annotated protein-coding genes were detected. All of these showed signals in the ribosomal footprinting assay indicating mRNA translation. Seven were verified by mass spectrometry. Fifty-seven genes are annotated in other enterobacteriaceae, mainly as hypothetical genes; the remaining 15 genes constitute novel discoveries. In addition, protein structure and function were predicted computationally and compared between EHEC-encoded proteins and 100-times randomly shuffled proteins. Based on this comparison, 61 of the 72 novel proteins exhibit predicted structural and functional features similar to those of annotated proteins. Many of the novel genes show differential transcription when grown under eleven diverse growth conditions suggesting environmental regulation. Three genes were found to confer a phenotype in previous studies, e.g., decreased cattle colonization.

**Conclusions:**

These findings demonstrate that ribosomal footprinting can be used to detect novel protein coding genes, contributing to the growing body of evidence that hypothetical genes are not annotation artifacts and opening an additional way to study their functionality. All 72 genes are taxonomically restricted and, therefore, appear to have evolved relatively recently *de novo*.

**Electronic supplementary material:**

The online version of this article (doi:10.1186/s12864-016-2456-1) contains supplementary material, which is available to authorized users.

## Background

EHEC is an important human pathogen, which causes bloody diarrhea, hemolytic uremic syndrome and other sequelae [1]. Strain EDL933 has been isolated from ground beef in Michigan (USA) and has been linked to the first severe EHEC outbreak in 1982 in which this bacterium was shown to be the causative agent [2].

Next generation sequencing (NGS), especially if conducted strand-specifically on mRNA (RNAseq), has grown into a valuable tool to study bacterial transcriptomes [3]. However, transcripts represent mRNAs as well as non-coding RNAs (ncRNAs), which sometimes are hard to tell apart. Especially, transcripts of small proteins might be mistaken for short ncRNAs [4–6]. A much better tell-tale for protein-coding RNA is the translatome consisting of ribosomal footprints, which indicate mRNAs being in the process of translation [7–10]. To determine the translatome, ribosomes are stalled on the mRNA and RNA parts accessible to RNase are degraded. The parts of mRNA covered by ribosomes are protected and form “ribosomal footprints” which are sequenced [10]. In addition, a high-throughput method to interrogate the bacterial proteome is the mass-spectrometry-based analysis of proteolytically digested proteins, usually referred to as “bottom up” or “shotgun proteomics” [11]. This method is able to directly confirm the existence of proteins, albeit displaying a limited sensitivity for low abundance proteins [12].

It is surprising that a sizable fraction of genes in any bacterium is still of hypothetical status, which means that their function is unknown and it is even unclear whether these genes are expressed [13]. Some hypothetical proteins have no homology to any other predicted protein in any species [14], i.e., they are taxonomically restricted [15] and, therefore, belong to the orphan genes. Other hypothetical genes are found in genomes of many species and are termed “conserved hypotheticals” [13]. Some hypothetical genes are believed to be annotation artifacts, suggesting that genomes are “over-annotated” and several hypothetical genes may not be protein-coding genes at all. For instance, after genome sequencing, genes are often predicted by computational annotation tools like GLIMMER [16]. These automatic tools might proliferate annotation errors. Yu et al. [17] state: “Previous works show that a significant fraction of annotated short ORFs may be not true genes, which is one of the major causes that account for the over-annotation of microbial genomes.” This reality has influenced the annotation of genomes from the release of the first eukaryotic yeast genome that began with over 9000 ORFs and shrunk to about 6000 when the authors realized a high error rate removing many ORFs shorter than 100 residues [18]. However, the above statements are based on publications from an era in which short proteins were largely dismissed as not functional, hence, not existing [19, 20]. Today, we control the technology to dig deeper: we add 72 short protein coding genes to the *E. coli* EHEC gene repertoire by using a multi-omics approach, including translatomic data and transcription patterns specific for different growth conditions.

### Methods

No ethics approval was required for any aspect of this study.

### Strain and culture conditions

The derivative WS4202 (Weihenstephan Microbial Strain Collection, Lehrstuhl für Mikrobielle Ökologie, Technische Universität München, Germany = CIP106327, Collection de l’Institut Pasteur, Paris, France) of *Escherichia coli* O157:H7 EDL933 (EHEC) was used in this study. Bacteria were incubated in 10-fold diluted lysogeny-broth [21] at 37 °C with shaking (180 rpm). The optical density of the samples was monitored at 600 nm to determine the growth phase. Before harvesting, an aliquot was plated on CHROMagar O157 (CHROMagar, France) to confirm identity. Cells were harvested by centrifugation (10,000×*g*, 1 °C, 3 min) in the transition from late exponential to early stationary phase. The pellet was frozen in liquid nitrogen and stored at -80 °C for RNA extraction. For ribosomal footprints, corresponding transcriptomes, and mass spectrometry, the strain was also grown in 10-times diluted LB. For the condition LB-Nitrite, 200 mg/L sodium nitrite was added and the pH adjusted to 6 using HCl. Briefly, bacteria were grown in LB medium at pH4, pH9, or at 15 °C; in LB with nitrite or trimethoprim-sulfamethoxazole; in LB-agar surface, M9 minimal medium, spinach leaf juice, surface of living radish sprouts, or in cattle feces. Further details about the growth conditions are described in [9].

### RNA-footprints and transcriptomes

RNA-footprints and transcriptomes (two biological replicates in each case) were analyzed as described by Landstorfer et al. [22] using 170 μg/mL chloramphenicol to stall the ribosomes, which is about 6-times above the concentration at which trans-translation occurs [23]. Isolated ribosomes were incubated with RNase I. Intact ribosomes were enriched twice by gradient centrifugation. Isolated footprints and isolated total RNA for transcriptome sequencing (rRNAs removed) were processed strand-specifically with the TruSeq Small RNA Sample Preparation Kit (Illumina) according to the manual and sequenced on an Illumina MiSeq.

Illumina FASTQ files were mapped to the genome using Bowtie [24] either in Galaxy [25, 26] or as standalone with default settings. Output SAM files were filtered for mappable reads using Samtools and further converted and indexed to BAM and BAM.BAI files [27]. Visualization of the data was carried out using our own NGS-Viewer [28] or BamView [29] implemented in Artemis 12.0 [30]. Transcription and translation levels were evaluated using RPKM values [31]. The RPKM value is defined as “reads per kilobase-gene length per million mapped reads”. In our calculations, we excluded rRNA reads since this value is compromised by the rRNA removal. All new open reading frames (ORFs) suspected of being translated (at least 10 RPKM translatome) were inspected manually [28] to exclude false positives, and the ribosomal coverage value (RCV) was calculated. The RCV is defined as the RPKM ratio of the translatome per transcriptome for each gene [31], both derived from the same biological experiment [22].

### Protein isolation

Cells were harvested by centrifugation at 4 °C for 4 min at 10.000 × *g* and washed five times with cooled Ringer solution. The pellet was resuspended in cold lysis buffer (50 mM Tris/HCl pH 7.5, 5 % glycerol, 1.5 mM MgCl_2_, 150 mM NaCl, 1 mM Na_3_VO_4_, 25 mM NaF, 0,8 % NP40/Igepal, 1 mM DTT and 1 tablet / 25 mL buffer of EDTA-free tablets of the protease inhibitor cocktail; Roche Diagnostics). Cells were disrupted using a Fast-Prep (MP Biomedicals) with six runs for 20 s each at a shaking speed of 5 m/s and cooling on ice between each run. The suspension was then incubated on ice for 30 min and subsequently centrifuged for 10 min at 20.000 × *g* at 4 °C. The supernatant was transferred to an ultracentrifuge tube and centrifuged 1.5 h at 4 °C at 141,000 × *g* in a Beckmann L7 ultracentrifuge. The supernatant was transferred into fresh tubes and stored at −80 °C. Protein concentration was determined by Bradford assays (RotiQuant, Roth), measuring extinction on a Victor^3^ 1420 multilabel counter (Perkin Elmer). The standard curve was generated using BSA (bovine serum albumin). The lysate was fractionated by SDS gel electrophoresis into 12 fractions. Each gel-fraction was washed and digested with trypsin for mass spectrometry (MS) analysis.

For the protein fraction “LB-small”, small proteins were fractionated by separating 300 μl of the above protein solution on two SDS gels. The proteins below 12 kDa were excised from the gel and transferred to a 0.5 ml microcentrifuge tube, which was pierced with a 20-gauge needle at the bottom. This tube was placed in a 1.5 ml microcentrifuge tube and centrifuged for 2 min at 13,000 × *g* at room temperature. The resultant gel debris was transferred to 500 μl elution buffer, rotated over night at room temperature in a microcentrifuge tube and then filtered with a 0.22 μm Spin-X spin filter (Corning, USA) for 2 min at 10,000 × *g*. Next, the proteins were precipitated using four-volumes of cold acetone (−20 °C), incubated at −20 °C for 60 min and then centrifuged at 15,000 × *g* for 10 min at −20 °C. The supernatant was decanted and remaining liquid evaporated at room temperature. Finally, the small protein fraction was treated with trypsin for MS analysis.

### Mass spectrometry

The digested protein fractions were subjected to an Eksigent nanoLC-Ultra 1D+ (Eksigent, Dublin, CA) coupled to an Orbitrap Velos (Thermo Scientific, Bremen, Germany). Peptides were delivered to a trap column (100 μm inner diameter × 2 cm, packed with 5 μm C18 resin, ReproSil-Pur AQ (Dr. Maisch, Ammerbuch, Germany) at a flow rate of 5 μl per min in 100 % buffer A (0.1 % formic acid in HPLC-grade water). After 10 min of loading and washing, peptides were transferred to an analytical column (75 μm × 40 cm C18 column, ReproSil-Pur AQ, 3 μm, Dr. Maisch) and separated using a 110-min gradient from 2 % to 35 % of buffer B (0.1 % formic acid in acetonitrile) at a 300 nl per min flow rate. Full-scan mass spectrometric spectra were acquired in the Orbitrap at mass resolution of 30,000. The five most intense precursors were selected for HCD fragmentation (isolation width, 2.0 Th) with a normalized collision energy of 40 % at an AGC target setting of 50,000. HCD spectra were acquired in the Orbitrap at a mass resolution of 7,500. Dynamic exclusion was enabled for a 10-s repeat duration and a 10-s exclusion duration with a repeat count of one. The MS results were based on three biological experiments including LB-standard, LB-nitrite and LB-small.

Raw mass spectrometric data files were converted into Mascot generic format files (MGF) using Mascot Distiller (2.4.2.0, Matrix Science). The MGF files were searched against the *Escherichia coli* O157:H7 EDL933 non-redundant NCBI database (version 03.05.2011) and the six-frame translated genome (NC_002655) using the Mascot search engine (2.3.1, Matrix Science). Mascot parameters were: an enabled decoy search using a randomized database; monoisotopic peptide mass (considering up to two ^13^C isotopes); trypsin/P as protease; a maximum of two missed cleavages; peptide charges +2 and +3; peptide tolerance ± 5 ppm.; MS/MS tolerance ± 0.005 Da; instrument type ESI-Trap; fixed modification: carbamidomethyl (cysteine) and variable modification: oxidation (methionine).

The results from Mascot were further processed with the software Scaffold [32] for statistical validation and better visualization (parameters used were: peptide probability ≥ 80.0 %, protein probability ≥ 99.0 %, minimum two peptides resulting in zero hits for decoys in peptide spectra or protein. The identified proteins and peptides were visualized and investigated using Artemis [30].

### Computational biology

The search engine PlatProm [33] was used to find potential promoters nearby the candidate genes. PlatProm scores were calculated for each nucleotide in the genome to estimate the probability for being the starting point of transcription. Scores exceeding the background level by four standard deviations (SD; score ≥ 7.44) were considered as statistically significant (p < 0.00004). While most bacterial promoters are located within the 250 bp region upstream of the initiation codon, about 10 % of the transcription start sites are within a more upstream region of 250–650 bp from the start codon [34]. Therefore, we searched promoters within 650 bp upstream of the start codons of the ORFs. The positio﻿n with the highest potentiality to initiate transcription within this range was taken.

Homologues protein and gene sequences were searched using blastp and tblastn, respectively [35]. PredictProtein [36, 37] was used to generate predictions of protein functional and structural features. In particular, the following tools were applied: PROFphd (secondary structure and solvent accessibility, [38]), PROFtmb (transmembrane strands, [39]), TMSEG (transmembrane helices) and COILS (coiled-coil regions, [40]), ScanProsite (functional motifs, [41]), HMMER (PFAM domains, [42]), SomeNA (protein, DNA and RNA binding sites, [37]), PSI-BLAST [43] and HHblits (homology to known proteins, [44]), SEG (low- and high-complexity regions, [45]), ConSurf (evolutionary conservation of amino acids, [46]), DISULFIND (disulfide bonds, [47]). For disordered region predictions, PROFbval [48], UCON [49] and METADISORDER [50] were used. Further, PROFtmb (bacterial transmembrane β-barrels, [51]), Metastudent (Gene Ontology terms, [52]), and LocTree3 (subcellular localization, [53]) were applied. SignalP4.1 was used for the prediction of signal peptides [54]. In all cases, default settings were used.

In order to check whether the functional and structural features of the 72 novel proteins resemble those of known annotated proteins, we assembled a positive set of “real proteins” by randomly choosing four length-matched annotated EHEC-proteins for each of the 72 novel proteins. Of 288 proteins, one was dropped later since it was duplicated in the genome. The negative comparison set was generated by shuffling each of the 287 annotated comparison proteins 100-times, i.e. generating 100 new random sequences with the same amino acid distribution as in the original sequence, but destroying any positional signal. PredictProtein was applied to protein sequences of both sets (i.e. “real” and “shuffled”) and the result was then provided to the Support Vector Machine (SVM, [55]) implementation of WEKA [56] and the Radial Basis Function [57] to discriminate automatically between proteins of both sets. The SVM was trained on features predicted by PredictProtein for 287 annotated (positive data) and 2870 shuffled (negative data) protein sequences. A similar negative set of shuffled proteins for the 72 novel proteins was generated in the same way as for the 287 annotated proteins. The trained SVM (“real” versus “shuffled”) was applied to classify each of the 72 novel and corresponding 7200 shuffled novel proteins. The total sets of 100 shuffled proteins for the 72 novel and the 287 annotated proteins were used to calculate error bars for the predicted protein features.

Repeat sequences of X002 were detected using REPFIND [58] and its RNA was folded with mfold [59], both used with default parameters.

## Results

### Ribosomal footprinting reveals 72 novel short protein-coding genes

**Fig 1:**
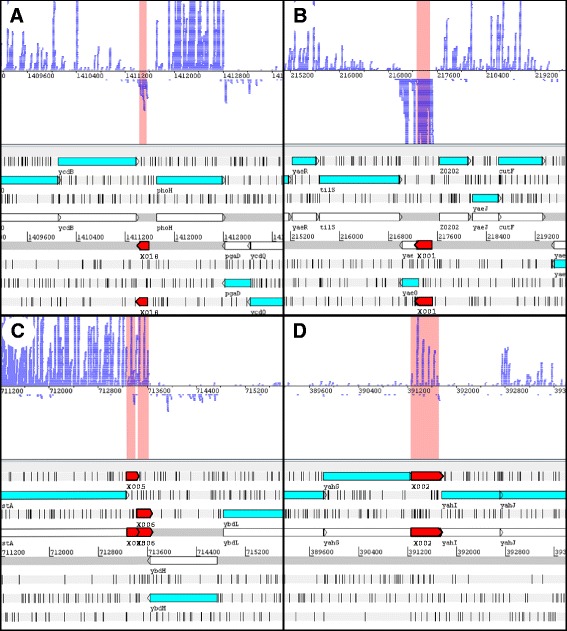
Four examples of new EHEC protein-coding ORFs (red arrows) discovered by ribosomal footprinting and visualized using Artemis [30]. Protein-coding ORFs are indicated by cyan arrows in the lower part of each panel. Blue lines in the upper part of each panel represent ribosomal footprint reads. **a** X018 is an example for a single (monocistronic) gene. **b** X001 is located in the upstream part of *yaeO*. These two genes might form a translationally coupled operon. **c** Two short genes, X005 and X006 are located downstream of *cstA*, maybe also translationally coupled. **d** X002 might be part of the operon *yahDEFGIJ* spanning from *yahD* to *yahJ* (only partly shown). The missing gene *yahH* had been annotated at first but was rejected later due to its structure (see Discussion and Fig 4)

We performed ribosomal footprinting which detects only RNA covered by ribosomes, i.e., mRNA. All intergenic, non-annotated ORFs of at least 153 bp (≥50 aa) were extracted from the translated-mRNA data set if a minimal threshold of 10 RPKM for the translatome was reached. This value is about 10-fold above background [9]. Each ORF of this subset was visually screened for its translational signal in the translatome to exclude false positives (e.g., translation of preceding or subsequent genes). This procedure yielded 72 previously un-annotated ORFs with an RPKM translatome between 13 and 2974. The mean for this value was 327 comparing to 404 of all annotated genes [not shown; 22]. Similarly, the ribosomal coverage value (RCV) was between 0.02 and 3.6 (Table 1) for the novel genes with an average of 0.9. The average RCV of annotated genes in this experiment was about 1.1 [not shown; 22]. Thus, the novel proteins are produced in lower abundance compared to annotated genes. In Fig. 1, the ribosomal footprinting pattern of four examples is shown in detail. The mRNA of the 72 genes was under translation to various degrees (Table 1) and, therefore, the genes received tentative gene names starting from X001. This labeling indicates that they are of unknown function, although their differential expression under diverse conditions was determined and for some a phenotype was found (see below). Most of the 72 new genes were short (≤315 bp, mean 210 bp), but three were longer (384, 465, and 804 bp).

**Table 1:** Novel genes detected in EHEC

Gene description						Ribosomal footprints^*e*^			MS^*h*^			PlatProm prediction^*i*^	
Name^*a*^	Classification^*b*^	Start^*c*^	Stop^*c*^	Length [bp]	Origin^*d*^	RPKM	Gene coverage^*f*^	Ribosomal coverage value^*g*^ (RCV)	LB	LB-Nit	LB-small	Upstream of start codon [bp]	Score
X001	real	217270	217488	219		1690	0.99	2.35				−460	9.00
X002 *yahH*	real	391261	391725	465		56	0.61	0.64				−211(*yahF*)	8.93
X003	real	570516	570710	195		102	0.72	0.69				--	--
X004	real	667557	667805	249		18	0.59	0.51	2		2	−287	7.90
X005	real	713269	713421	150		190	0.92	0.89				−54(*cstA*)	7.61
X006	real	713433	713630	198		166	0.86	0.77				−54(*cstA*)	7.61
X007*		790488	790682	195		79	0.65	0.80				−563	9.15
X008	real	902889	903083	195	phage	678	0.71	0.82				−14	7.63
X009*	real	978607	978747	141		17	0.52	0.50				−129	7.63
X010a	real	1112292	1112471	180		35	0.75	0.71				−297	8.28
X010b		1508079	1507899	duplicate of X010a									
X011*	real	1146872	1147027	156		13	0.53	0.38				--	--
X012	real	1152583	1152795	213		57	0.51	0.42				−2	7.66
X013	real	1256680	1256967	288	phage	230	0.89	0.92			2	−590	7.73
X014a	real	1267635	1267820	186	phage	552	0.66	0.26				−67	8.07
X014b		2314896	2314711	duplicate of X014a									
X015	real	1334776	1334931	156	phage	35	0.84	0.32				−70(*trxB*)	7.84
X016a	real	1346825	1347184	360	phage	58	0.65	0.69				−20	9.29
X016b		3000443	3000802	duplicate of X016a									
X017	real	1353605	1353772	168	phage	23	0.52	0.21				−91	9.27
X018	real	1411438	1411557	120		49	0.8	0.37				−30	10.92
X019	real	1680779	1680967	189	phage	242	0.77	3.51				−269	9.01
X020	real	1772962	1773144	183		53	0.6	1.04				−24(*dadA*)	10.52
X021	real	1843458	1843622	165		1029	0.65	2.56				−625	7.74
X022*	real	1866296	1866505	210	phage	2169	0.82	0.73				−6	7.63
X023*		1866493	1866648	156	phage	280	0.88	1.30				−203	7.63
X024	real	1881598	1881819	222	phage	21	0.37	0.60	2	2	2	−76	8.93
X025a		1389500	1389288	duplicate of X015b									
X025b	real	1888594	1888806	213	phage	524	0,95	0,08				−112	8,27
X026	real	1905731	1905850	120	phage	622	0.7	1.01				−77(Z2121)	12.18
X027	real	2038161	2038382	222		75	0.51	1.46				−313	7.47
X028*		2101101	2101247	147		131	0.61	1.70				0	8.00
X029	real	2109655	2109921	267		629	0.97	0.85				--	--
X030	real	2138823	2139137	315	phage	1520	0.98	1.35				−53	16.96
X031	real	2168349	2168567	219		77	0.66	0.60				−110	8.48
X032a		1269797	1269913	duplicate of X032c									
X032b		1868589	1868705	duplicate of X032c									
X032c	real	2312618	2312734	117		650	0.81	0.74				−447	7.83
X033*		2379507	2379659	153		348	0.87	1.50				−77	12.26
X034	real	2430386	2430598	213		47	0.53	0.22				−9	11.59
X035*		2480019	2480177	159		25	0.52	0.20				−63	10.51
X036	real	2584677	2584847	171		52	0.66	0.17				−162	12.18
X037	real	2663871	2664122	252		14	0.53	0.58				−243	12.65
X038	real	2670869	2671075	207	phage	1209	0.8	0.69				−28	11.39
X039	real	2742703	2742918	216		90	0.58	0.61				−103	7.60
X040	real	2777135	2777347	213	phage	37	0,57	0,02				--	--
X041	real	2779284	2779508	225	phage	57	0.73	1.32				--	--
X042	real	2844454	2844606	153		768	0.84	0.83				−295(X043)	8.26
X043	real	2844640	2844804	165		212	0.92	0.44				−295	8.26
X044	real	2844865	2845074	210		36	0.53	0.17				−210	11.00
X045	real	2845149	2845358	210		163	0.9	0.16				−23	9.54
X046*		2845408	2845602	195		145	0.69	0.35				−33	9.54
X047	real	2966787	2966987	201	phage	34	0.71	0.17				−21	8.08
X048	real	3003688	3003945	258	phage	40	0.65	1.96				−353	8.18
X049	real	3004951	3005067	117	phage	241	0.75	1.39		3	2	−93	9.71
X050	real	3013440	3013694	255	phage	28	0.64	0.47				−71(Z3371)	8.46
X051	real	3261588	3261758	171		89	0.86	0.35				--	--
X052*		3271689	3271820	132		34	0.79	0.32				−95	9.93
X053 *suhB*	real	3453780	3454583	804		41	0.53	0.20	9	13	2	−36	9.48
X054*		3894853	3894993	141		98	0.86	0.56				−220	8.25
X055	real	3918141	3918344	204		47	0.56	0.31				--	--
X056	real	4207372	4207641	270		725	0.92	0.66				−52	10.58
X057	real	4240665	4240883	219		2974	0.88	2.01				−24	13.80
X058*		4441485	4441643	159		359	0.98	0.64				−569	9.75
X059	real	4449723	4449821	99		19	0.6	0.08				−96	7.97
X060	real	4468299	4468592	294		639	0.84	2.99				−253	9.57
X061	real	4585965	4586174	210		202	0.92	1.98		2	2	−67	9.03
X062	real	4654347	4654490	144	phage	29	0.73	0.89				−393	8.17
X063*		4730352	4730537	186		15	0.51	0.95				−533	11.48
X064	real	4793504	4793737	234		20	0.53	0.28				--	--
X065	real	4870817	4870978	162		38	0.74	1.28				−90(*pldA*)	8.1
X066*		4873916	4874122	207		117	0.84	2.58				−104	7.92
X067	real	4916583	4916756	174		162	0.84	0.64				−22(*yihI*)	11.84
X068*		5077694	5077831	138		2040	0.97	0.55				−368(*nfi*)	7.61
X069	real	5369765	5369998	234		141	0.94	0.33				−159(*pepA*)	11.47
X070	real	5456776	5457042	267		53	0.52	3.58				−163(*yjiM*)	8.02
X071	real	5494158	5494394	237		45	0.57	2.82				−27	8.35
X072	real	5515374	5515541	168		38	0.69	0.80				−39(*serB*)	7.9

^*a*^ The asterisk indicates genes not annotated in any other organism (blastp against GenBank, threshold E-value ≤10^−10^)

^*b*^ Machine learning classification based on the set of annotated proteins (“real”) and their shuffled counterparts as training set

^*c*^ The positions are given in relation to GenBank accession no. NC_002655, the original genome sequence of strain EDL933. Only very recently, the genome has been updated (GenBank accession no. CP008957)

^*d*^ Genes originating from prophages are indicated

^*e*^ The RPKM footprint and coverage of the actual ORF with footprints is given as average of two replicate experiments for bacteria grown in LB medium

^*f*^ Fraction of the ORF covered with one or more footprint reads

^*g*^ Ratio of RPKM footprints to RPKM transcriptome

^*h*^ Indicated is the number of individual peptide spectra gained by mass spectrometry

^*i*^ Putative promoters have been predicted using PlatProm. The position of the assumed transcription start site upstream of the start codon and the quality of the prediction (score) are given

### Bioinformatics analysis of the proteins encoded in the novel genes

Suitable σ^70^-dependent promoters were predicted by PlatProm within the potential regulatory region of 50 candidate genes, while additional 14 genes are possibly transcribed as polycistronic units together with upstream genes (Table 1). Most novel genes, therefore, appeared to be driven by the housekeeping form of the RNA polymerase [34].

Protein sequences of the 72 new genes were submitted to PredictProtein [36], a powerful protein-analysis tool, which provides predictions of various aspects of protein structure and function (see Material & Methods and Additional file 1). The goal of this study was to compare the newly discovered proteins (“novel”) at a broader scale with gene products of annotated genes. Towards this end, a random choice of length-matching annotated proteins from EHEC was used as a control (“annotated”). To exclude excessive bias using randomly chosen annotated proteins, each novel protein of the 72 was length-matched with four annotated gene products.

**Fig 2:**
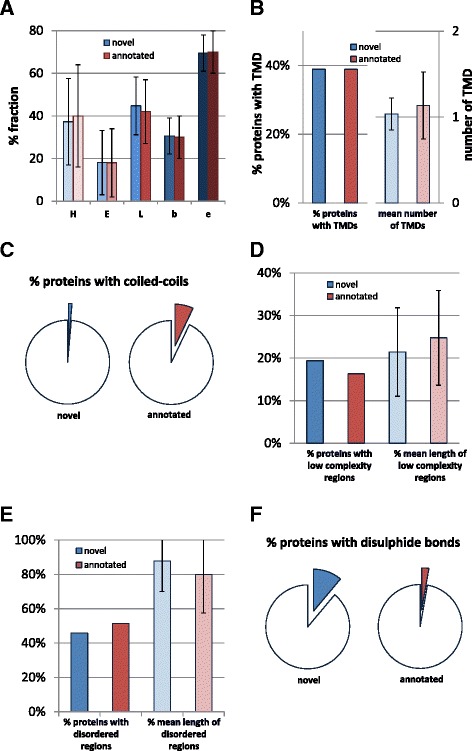
Graphical overview of PredictProtein values for the novel and length-matched annotated proteins. Error bars (if given) show the SD. **a** Shown is the predicted percentage of the protein length comprised of helices H, sheets E, and loops L. Furthermore, the percentage of buried and exposed amino acids is given (b and e). **b** On the left side, the fraction of proteins possessing at least one predicted transmembrane domain (TMD) is shown. On the right side, the mean number of TMDs per possessing proteins is shown. **c** The fraction of proteins having a coiled-coil prediction using a window of 14 amino acids is given. **d** The left bars show the fraction of proteins with a low-complexity region, the right bars give the mean length of this region compared to the overall length of the proteins for those possessing such a region. **e** The left bars show the fraction of proteins with a disordered region, the right bars give the mean length of this region compared to the overall length of the proteins for those possessing such a region. **f** The fraction of proteins having at least one Cys = Cys bond predicted

The secondary structure prediction (helix, H; beta-sheet, E; loop, L; Fig. 2a) did not show any conspicuous difference between novel and annotated proteins. This was also true for the percentage of buried (b) versus exposed (e) residues (Fig. 2a). About 40 % of the proteins in both groups were predicted to contain transmembrane helices (mostly single-span membrane proteins, Fig. 2b). Furthermore, only one protein (≈1.4 %) from the set of 72 novel proteins had a predicted coiled-coil (of 14 residues), compared to 8 % of the annotated proteins (Fig. 2c, Additional file 1).

Low complexity segments circumscribe protein areas of “low information content” [45, 60]. Of the novel proteins, 19 % contained low complexity regions versus 16 % of the annotated. In the novel protein group, these regions tended to be marginally shorter (on average about 21 % of the protein length) compared to the annotated proteins (on average about 25 % of their length), but their distributions overlapped largely, thus, the difference was insignificant (Fig. 2d). Disordered regions were counted if MetaDisorder predicted intrinsically disordered stretches of 30 or more consecutive residues. About 45 % of the novel proteins contained such a disordered region, encompassing on average 88 % of the protein length. In the control set, 51 % of the proteins contained a disordered region, encompassing on average about 80 % of the protein length. Thus, slightly fewer of the novel proteins possessed a disordered region, but these fewer regions tended to be slightly longer than those in the annotated proteins (Fig. 2e). β-barrels are generally rare in proteins and none was detected in both groups (not shown).

**Fig 3:**
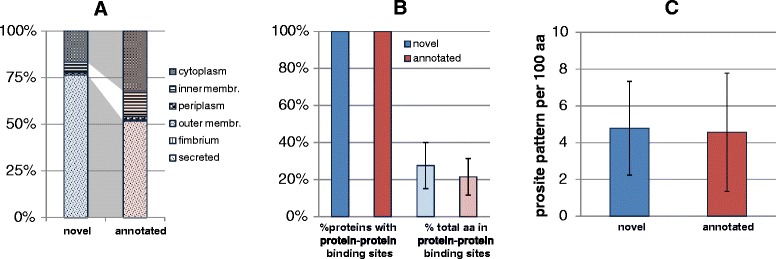
Graphical overview of the PredictProtein values for the novel and length-matched annotated proteins comparing localization, protein-protein binding sites, and PROSITE pattern. **a** Subcellular localization has been predicted using LocTree3 and is shown in per cent for the different compartments (membr., membrane). **b** The left bars show that all proteins have predicted protein-protein binding sites. The right bars show the percentage of the predicted number of amino acids involved in this type of interaction. **c** Given is the predicted number of PROSITE patterns per 100 aa

Interestingly, about 11 % of the novel proteins were predicted to contain disulfide bond-forming cysteine residues compared to 3 % in the control set of annotated proteins (Fig. 2f). The higher number was not explained by an over-representation of cysteine (which was observed but only an over-representation by a factor of 1.5, i.e. maximally explaining 2.25 times more disulfide bridges, not 3.6 times more). Instead, the high number of disulfide bridges might suggest an abundance of secreted proteins. To test if the single-span membrane helices found above might be signal peptides, we examined those proteins which have one predicted transmembrane region using SignalP [54] to discriminate between true transmembrane domains and signal peptides. We found a lower percentage of the novel proteins to possess a signal peptide (22 %) compared to the annotated proteins (33 %; Additional file 2). However, using LocTree3 that combines homology-based inferences with *de novo* predictions of sub-cellular localization [53], we found that the novel proteins contain a larger fraction of proteins predicted to be secreted than the annotated set: over 75 % proteins in the novel set and only about 50 % in the annotated group were predicted as secreted (Fig. 3a). This fits to the above observation of an over-representation of disulfide bridges, typical for secreted proteins.

All proteins were predicted to contain protein-protein binding sites under consideration of proximal residues, varying between 1 to 54 residues per protein. The percentage of residues predicted to be involved in protein-protein binding was slightly larger for the novel proteins than for the annotated (28 % vs. 22 %; Fig. 3b). ScanProsite predicted functional domains and motifs [41]. Since they are of various length, we determined how many PROSITE patterns [61] exist per 100 residues. Interestingly, both the novel and annotated proteins have almost five of such patterns (Fig. 3c).

ConSurf estimates the evolutionary conservation of residues based on the phylogenetic relations between homologous sequences [62]. We counted all residues with a ConSurf value of 5 or higher. Interestingly, there was virtually no difference between both protein groups (not shown). For some of the novel proteins detected, Gene Ontology (GO) terms were predictable using Metastudent [52]. Predicted functions included stress response, protein binding, transcription regulation and metabolic processes for X001, X006, X030, X042, X043, X044, X047, X053, and X061 (Additional file 3).

### Comparison of “real” versus “shuffled” protein sequences

To evaluate if real versus shuffled can be distinguished using computational predictions, we randomly shuffled each novel protein sequence as well as each annotated protein sequence from the comparison set 100-times. Structure and function features of the shuffled sequences were predicted with PredictProtein as before. For all features the mean  ± 1 SD for all 100 shuffled sequences was compared to the value for the native protein sequences (if applicable; raw data in Additional file 4). Surprisingly, real proteins and their shuffled counterparts superficially showed similar distributions for most predictions (Additional file 5) and several of the real proteins had values within the mean  ± 1 SD of their shuffled counterparts (Additional file 6). To gain further insight, we used machine learning to distinguish between 72 real and 7200 shuffled sequences. The machine was trained using the set of “real” proteins and their shuffled counterparts. Of 72 novel proteins, 61 (true positives; 85 %) were recognized as real, while of 7200 shuffled, only 8 (false positives; 1 %) were classified as such. Interestingly, all seven novel proteins with MS data (see below) were classified as “real” (Additional File 7).

### Environmental regulation of transcription under eleven different growth conditions

To check for specific transcription of the newly discovered genes in comparison to the standard LB medium, we analyzed transcriptome data of the strain grown under a variety of different conditions [9]. Briefly, the bacteria were grown in LB medium at pH4, pH9, or at 15 °C; in LB with nitrite or trimethoprim-sulfamethoxazole; on the surface of LB-agar, in M9 minimal medium, in spinach leaf juice, on the surface of living radish sprouts, or in cattle feces. We found specific transcription profiles for each gene in comparison to standard LB, e.g., X071 was only induced in minimal medium, X059 only on radish sprouts, and X031 only in spinach leaf juice. X062 was up-regulated in minimal medium, LB with nitrite, and LB pH9; while X060 was induced in spinach leaf juice or at 15 °C. Cow dung, LB agar surface, and LB at pH4 did not show up regulation of any of the new genes, but rather a down regulation of several of them (Table 2, Additional file 8).

**Table 2:** Transcriptome data of selected novel genes regulated under specific conditions given as fold-change compared to standard LB^*a*^. Data are taken from [9]

Name ^*b*^	Minimal medium	LB-Nit	pH9	Radish sprouts	Spinach leaf juice	15 °C	Amoeba	Antibiotics	Cow dung	Agar surface	pH4
X009*	u/c	n.r.	9	u/c	u/c	n.r.	70	u/c	u/c	n.r.	n.r.
X011*	12	u/c	6	8	26	13	151	n.r.	n.r.	19	21
X031	u/c	u/c	u/c	u/c	26	u/c	u/c	u/c	u/c	u/c	−18
X037	n.r.	n.r.	n.r.	n.r.	n.r.	n.r.	213	n.r.	n.r.	n.r.	n.r.
X048	n.r.	- u/c	u/c	u/c	n.r.	u/c	n.r.	48	n.r.	n.r.	u/c
X052*	n.r.	−6	−5	n.r.	u/c	u/c	12	n.r.	n.r.	n.r.	−5
X060	u/c	7	u/c	5	10	18	u/c	−17	u/c	u/c	u/c
X062	25	14	12	n.r.	u/c	7	23	n.r.	n.r.	n.r.	n.r.
X070	n.r.	n.r.	u/c	n.r.	n.r.	u/c	25	n.r.	n.r.	n.r.	n.r.
X071	122	14	9	5	u/c	u/c	n.r.	n.r.	5	n.r.	n.r.

^*a*^positive values, up regulated; negative values, down regulated; n.r., no reads under this condition; u/c, unchanged (threshold ≥5-fold regulation)

^*b*^The asterisk indicates genes not annotated in any other organism (see Table 1)

We further performed a transcriptome analysis of EHEC grown in the presence of amoeba (*Acanthamoeba castellanii*; data not shown). This experiment yielded not enough sequencing reads for a proper global comparison to the other conditions, but still allowed to deduce specific up regulation of transcription: > 10-fold compared to LB for X009, X011, X037, X052, X062, and X070. These results show that gene expression of the novel genes changes in a diverse array of conditions which might indicate functionality.

### MS data confirm expression of seven novel genes

When evaluating MS data of cells grown under the same conditions used for transcriptome and translatome analyses, as well as evaluating a six-frame translation of the EHEC-genome, we observed peptide signals belonging to seven of the new ORFs (Table 1).

### An REP-element containing ORF, X002, is translated

**Fig 4:**
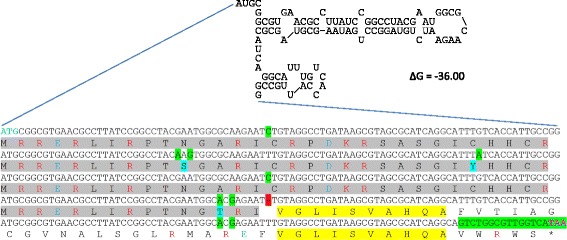
Repeat structure of the REP23 containing gene *yahH* (the same as X002) and its protein YahH [58]. The upper part shows one repeat block folded as mRNA [59]. The DNA sequence (lower part) basically consists of five of such repeated blocks, with only minor differences (when compared to each other – single nt differences are in green) and a short unique sequence at the 3’-end (green stretch). When comparing the fourth block to the other, a base appears to be missing (red marked gap) causing a change in the reading frame visible in the protein structure. Thus, the protein contains three large repeats and a fourth truncated one (grey blocks, few differences in aa indicated in blue). Downstream of the “frame shift” mutation, a different structure of two blocks is found (yellow). The protein contains many charged amino acids, either positive (RK, red print) or negative (DE, blue print)

While screening the genome for the novel genes, X002 piqued our specific interest (Fig. 1d), since it falls within a gap located between *yahG* and *yahI*. Presumably, this gene had been annotated as *yahH* but was removed later. X002 contains a sequence, which is a REP element, belonging to the group of bacterial interspersed mosaic elements (BIME). The amino acid sequence of X002 matched REP23 from *E. coli* K-12 in a blastp search [35] with an E-value of 3 × 10^−174^ [52, 63, 64]. The gene locus of X002 contains a long ORF (465 nt) and its transcript was well covered by ribosomal footprints (Fig. 1d). Using REPFIND [58], we discovered a block-like structure of five sequence repeats within the ORF. One of these repeated elements was predicted to fold in a relatively stable stem-loop structure according to mfold [59; Fig. 4]. The highly repetitive nature of this ORF was also visible in the footprint signal, which appeared to be very regular (Fig. 1d). The translation of such REP-elements is somewhat unexpected.

### Phenotypes of novel genes in cattle

Transposon (Tn) or signature-tagged mutagenesis (STM) studies of EHEC [65–74] were evaluated to find possible insertion mutants of the novel genes conferring a phenotype. Only few studies contained phenotypic data of intergenic insertions. We used a stringent threshold of 5-fold difference between the wild type and the transposon-mutated strain. Nevertheless, three novel genes could be linked to a phenotype in cattle: an existing EHEC-transposon mutant library [74] had been re-screened for mutants changed at least 5-fold in their ability to colonize the ruminant intestine [72]. These novel ORFs (i.e., X033, X036, and X045) were found to either have a direct transposon hit or a hit shortly upstream of its frame, hence, supposedly in their promoter region (Table 3). Transposon hits of genes X036 and X045 had already been detected in the original STM study looking only for a decrease in cattle colonization [74]. The finding that some of the novel genes display a phenotype in cattle is interesting, as it indicates our fragmentary knowledge about EHEC-host relationships and highlights the importance of short genes in general. However, future research must show if the changed colonization of the transposon-mutants is indeed caused by the novel genes hit or are only a byproduct of the transposon inserted in these positions.

**Table 3:** Phenotype in calves of transposon hits in or nearby the novel genes. Threshold is defined as a 5-fold or higher regulation. Negative values indicate down-regulation. Data are taken from [72]

Name^*a*^	Position of Tn insertion	Direct hit [H] or bases upstream [b]	Fold-change output versus input
X033*	2379421	86	−33
X036	2584780	H	−13
X045	2845234	H	−50

^*a*^The asterisk indicates genes not annotated in any other organism (see Table 1)

### Taxonomic distribution of the novel genes

For all 72 genes, homologous genome regions could be detected in *E. coli* O157:H7 Sakai using tblastn [35]. Twenty-eight of the 72 genes had been annotated in Sakai as protein-coding ORFs. Fifty-seven of the 72 genes were found to be annotated within the enterobacteriaceae using blastp (E-value threshold ≤10^−10^, E-value median for all top hits of the novel proteins was 5.5 × 10^−34^). Fifteen out of the 72 genes were unique which means that these had not been annotated as protein-coding genes before (marked by an asterisk in Table 1). Significantly, all 72 genes had no blastp (E-value ≤10^-10^) hit beyond the enterobacteriaceae in GenBank [75]. Thus, these genes appear to be taxonomically restricted to this single family of the order enterobacteriales, sometimes even to the species *E. coli*. Further, for 40 out of 72 proteins, the family members found using blastp in GenBank (40 genes) were exclusively labeled as “hypothetical” or “conserved hypothetical”. We substantiated this trend using various thresholds for defining families with PSI-BLAST [43] and HHblits [44]. For instance, at PSI-BLAST E-values ≤10^−3^ and HHblits E-values ≤10^-10^, about 40 % of the 72 novel genes were found to be novel (i.e., orphans), while the corresponding fraction for the control set was at least 20-times smaller (<2 %). Another 10–20 % had families smaller than the corresponding control set. However, the remaining 40–50 % of the 72 had families of similar sizes as the proteins in the control set (Additional file 9). Sixteen of 72 proteins had, at least to some extent, a functional annotation and 21 were of prophage origin (see Additional file 8).

## Discussion

### The short hypothetical genes are no annotation artifacts

Although *E. coli* is probably the bacterial species researched best, many environmental growth conditions have never been evaluated and many cannot be evaluated easily in the laboratory. This fact may partly explain why a sizable fraction of genes in any bacterium is still of hypothetical status. “Our lack of fundamental knowledge about the function of so many of the building blocks of cells“, as stated by Roberts [76], hampers downstream research and other –omics efforts [77], since only what is known will be examined. Unfortunately, the smaller the protein-coding ORFs, the more likely it is that such genes are either ignored based on the assumption that short ORFs are highly unlikely to be functional [19, 78], not predicted due to the bias towards longer ORFs [79] or evade detection due to technical difficulties [20, 80]. In addition, many of the novel proteins are supposedly secreted according to our LocTree3 data and, thus, may be missed by the proteomics approach.

Ribosomal footprinting provides a high-throughput method, which indicates that also short genes encode proteins, as a footprint fragment (i.e., a nucleic acid) can be detected much easier than a short protein [81] and independent of its final destination within or outside the cell. The mRNA of the novel genes described here was clearly covered by ribosomes, thus indicating translation [82, 83]. This hypothesis was corroborated by bioinformatics analysis: only few of the general protein structure and function traits were predicted to differ between the novel 72 proteins described here and a set of annotated proteins with similar length distributions. The exceptions were disulfide bonds and coiled-coils, but these two parameters mattered only for a small subset of the proteins. The largest difference was obtained in the predicted sub-cellular protein location using LocTree3. Interestingly, when examining the “dark proteome” – that is proteins never observed by experimental structure determination and, therefore, inaccessible to homology modeling, similar trends were observed [84]. For instance, these proteins were short, often secreted, and had a higher amount of disulfide bonds [84]. However, in most parameters investigated, differences appeared to be minor. This was corroborated by a machine learning approach, able to classify 61 of the 72 novel proteins to resemble known annotated proteins but not random sequences. These results suggest that the protein sequences encoded by the novel genes described here show the same structural features and, thus, functional traits as well-known annotated proteins [85], validating the idea that such sequences form the raw material for evolutionary optimization of novel proteins [86].

We found that several of the new genes are specifically induced only under one or a few growth conditions hardly ever tested in the lab. According to Hemm et al. [4] and Hobbs et al. [87], short proteins seem to be important for the stress response of *E. coli*. Indeed, the novel genes discovered in this study were found to be induced under specific and sometimes adverse culture conditions such as minimal medium, pH9, radish sprouts, spinach leaf juice, antibiotics, cow dung, and the presence of amoeba (Table 2, Additional file 8). Furthermore, transposon-mutants derived in a previous study conferred a phenotype for three novel genes detected. The mutants had a decreased ability to colonize the cattle intestine [72]. The fact that genes detected in our study were connected to a phenotype in a cattle study shows that not only well-known genes of “standard properties” play an important role in the bacterial life cycle, but also such short novel proteins, maybe as a toxin [88]. Indeed, we predicted an unusual abundance of secretion in the set of 72 novel proteins (ca. 75 % secreted proteins, Fig. 3a).

Seven out of 72 genes were validated by proteome analysis. However, the probability to detect a protein via MS decreases with the size of the protein. Peptide fragments between 7 and 13 residues have the highest probability of detection, whereas fragments below 5 or above 40 residues are missed [89]. Short proteins are less likely to be detected by MS due to possibly missing tryptic cleavage sites. The tryptic cleavage sites typically occur C-terminally of an arginine (R) or lysine (K). If none of these amino acids is present, no fragmentation occurs and the peptides are too long for successful detection [90]. No R or K are found in five of the novel proteins, but none of the annotated. Thus, not surprising, most MS spectra originated from the largest of the 72 proteins. In addition, the probability to detect a protein is strongly dependent on its abundance [91], but the novel proteins are less abundant proteins (lower RPKM and RCV values compared to the annotated).

Based on the multi-omics approach by combining data of transcriptomes, translatomes, mass spectrometry, bioinformatics analyses and phenotype searches, we suggest that these 72 short genes are an overlooked fraction of genes in the EHEC genome, which should be added to the genetic map of this bacterium. We showed that even densely covered genomes like those of bacteria (in which about 90 % of the genome is covered by annotated protein-coding genes) still provide room for new protein-coding genes. This finding also adds to the growing evidence that even short hypothetical genes of bacterial genomes are no artifacts [19]. However, there might be coding sequences for even shorter polypeptides [our unpublished data; 78]. Their detection is at the resolution limits of most experimental techniques and only targeted multi-omics approaches may resolve the problem in the future.

### *yahH* – a gene locus with a potential triple function?

The novel gene X002 turned out to be a REP-element belonging to the bacterial interspaced mosaic elements. These elements play several roles based on their repetitive DNA sequence [64]. They are believed to be topological insulators for transcription-induced positive supercoiling and may bind proteins such as IHF, PolI and DNA gyrase to structure the DNA [63, 64]. Further, such elements can initiate a Rho-dependent transcription attenuation [92] and may stabilize RNA by inhibiting its degradation in vivo [93, 94]. Therefore, Gonnet et al. [95] suggested that it is highly unlikely that the REP-element *yahH* is translated, and, consequently, this gene was removed from the annotation [52] based on its unusual gene structure (Fig. 4). Interestingly, the Rho-dependent transcription attenuation of REP-elements is abolished, if the repeat element is translated [92]. Using our assay, we could show that X002, which is equivalent to *yahH*, is not only transcribed, but quite probably also translated. The resulting protein has a high number of charged amino acids and is of unknown function. If true, this gene locus would carry a triple function, i.e., as regulatory DNA element, as a regulatory RNA element, and in addition, as a protein.

### The novel genes evolved recently

All novel genes described here are restricted to the enterobacteriaceae or even to taxa closer related, and, therefore, are taxonomically restricted genes (TRGs [96]). They appear only in higher phylostrata (i.e., closer relatives; [97]), which is evidence for their relatively recent origin [98]. This hypothesis is supported by several findings: the novel genes described here apparently use more often one of the rare start codons (e.g., GTG, TTG), which are translationally less optimal [99] and, therefore, may not yet be evolutionarily optimized. In addition, the novel genes are clearly shorter than the average *E. coli* genes and some are not classified as “real” by our bioinformatics approach. All of the above corroborates the findings of Tautz & Domazet-Lošo [15], who also observed that TRGs are generally shorter than conserved genes and confer weak phenotypes. Most of the newly discovered proteins are located directly up- or downstream of annotated, “established” genes (compare to Fig. 1b-d), perhaps contributing a (minor?) constituent to already known operons [19]. This particular arrangement may indicate a potential evolutionary mechanism to sample genetic regions, which may form a coding reserve, i.e., short ORFs are tested for their usefulness for the cell. A minor upstream promoter activity (or a weak terminator site) would permit the formation of polycistronic RNA carrying additional ORFs upstream (or downstream, respectively). Thus, ribosomes may bind “too early” or ribosomes which are already bound to the mRNA while translating an established upstream gene will initiate and translate a downstream short ORF with a higher probability, respectively. If the resulting protein provides a significant fitness gain, it may promote strain survival and subsequent improvements by classical Darwinian evolution.

## Conclusion

It has been suggested by Carvunis et al. [86] that genes form *de novo* from non-coding DNA in yeast. Although prokaryotes possess much less non-coding intergenic DNA due to their dense gene content, such a mechanism as detailed above might be active in bacteria as well. However, the DNA sequence features which would allow for the formation of a protein sequence functional *ab initio* upon accidental expression of an intergenic, non-protein coding DNA sequence (i.e., being of supposedly random amino acid content) remain unknown [100].

### Availability of supporting data

All additional files supporting the results of this article are available in the repository labarchives.com (http://www.labarchives.com/) using the link http://dx.doi.org/10.6070/H4610XB9. All the supporting data are included as Additional files.

## Additional files

Additional file 1:
**Summarized values of PredictProtein for the novel proteins and length-matched annotated proteins.** (XLSX 81 kb) Additional file 2:
**SignalP prediction of signal peptides for proteins with one transmembrane domain.** (PDF 74 kb) Additional file 3:
**Gene Ontology terms predicted using Metastudent for the novel genes.** (XLSX 12 kb) Additional file 4:
**Raw data of PredictProtein for all natural sequences (72 novel and 288 annotated) and their 100-times shuffled counterparts.** (XLSX 16697 kb) Additional file 5:
**Prediction values for the novel and annotated proteins compared to their shuffled counterparts in dependence of the protein length.** (PDF 443 kb) Additional file 6:
**Prediction values for the novel proteins compared to their shuffled counterparts for each protein individually.** (PDF 144 kb) Additional file 7:
**Overview of the machine learning results.** (XLSX 10 kb) Additional file 8:
**Expanded data set for all novel genes.** (XLSX 56 kb) Additional file 9:
**Cumulative distributions of PSI-Blast and HHblits family sizes.** (PDF 8541 kb)

## Abbreviations

AA: amino acids
AGC: automatic gain control
b: buried or base
BLAST: basic local alignment search tool
BIME: bacterial interspersed mosaic elements
BP: base pairs
BSA: bovine serum albumin
e: exposed
E: beta-sheet
EHEC: enterohemorrhagic *E. coli*
GO: gene ontology
H: helix
HCD: Higher-energy collisional dissociation
HPLC: high pressure liquid chromatography
kDa: kilo Dalton
L: loop
LB: Luria-Bertani medium
membr: membrane
MGF: Mascot generic files
MS: mass spectrometry
ncRNA: non-coding RNA
NGS: next generation sequencing
n.r.: no reads
ORF: open reading frame
RCV: ribosomal coverage value
REP: repetitive extragenic palindrome
RNAseq: mRNA sequencing
RPKM: reads per kilobase per million mapped reads
RPM: revolutions per minute
SD: standard deviation
SDS: sodium dodecyl sulfate
STM: signature-tagged mutagenesis
SVM: support vector machine
TMD: transmembrane domain
TN: transposon
TRG: taxonomically restricted genes
u/c: unchanged
