# Supplementary material for: Translatomics combined with transcriptomics and proteomics reveals novel functional, recently evolved orphan genes in Escherichia coli O157:H7 (EHEC)
Source: BMC Genomics. 2016 Feb 24;17:133. doi: 10.1186/s12864-016-2456-1 (PMC4765031; doi:10.1186/s12864-016-2456-1)
Supplement: Additional file 2: — SignalP prediction of signal peptides for proteins with one transmembrane domain. (PDF 74 kb) [file 12864_2016_2456_MOESM2_ESM.pdf]

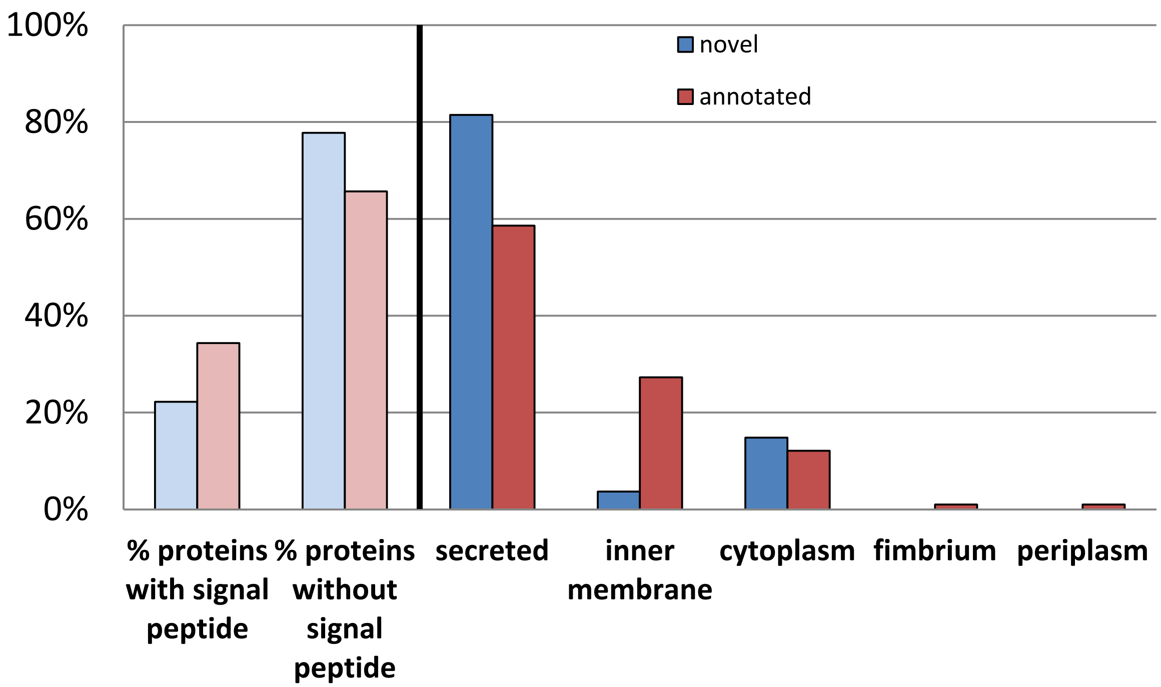

(Left part) Graphical overview of novel and length-matched annotated proteins either possessing a signal peptide detected using SignalP or not. **(Right part)** Predicted subcellular localization using LocTree3. Note, that for the analysis shown here only proteins with one predicted transmembrane-domain (TMD) were considered.
