# Supplementary material for: Translatomics combined with transcriptomics and proteomics reveals novel functional, recently evolved orphan genes in Escherichia coli O157:H7 (EHEC)
Source: BMC Genomics. 2016 Feb 24;17:133. doi: 10.1186/s12864-016-2456-1 (PMC4765031; doi:10.1186/s12864-016-2456-1)

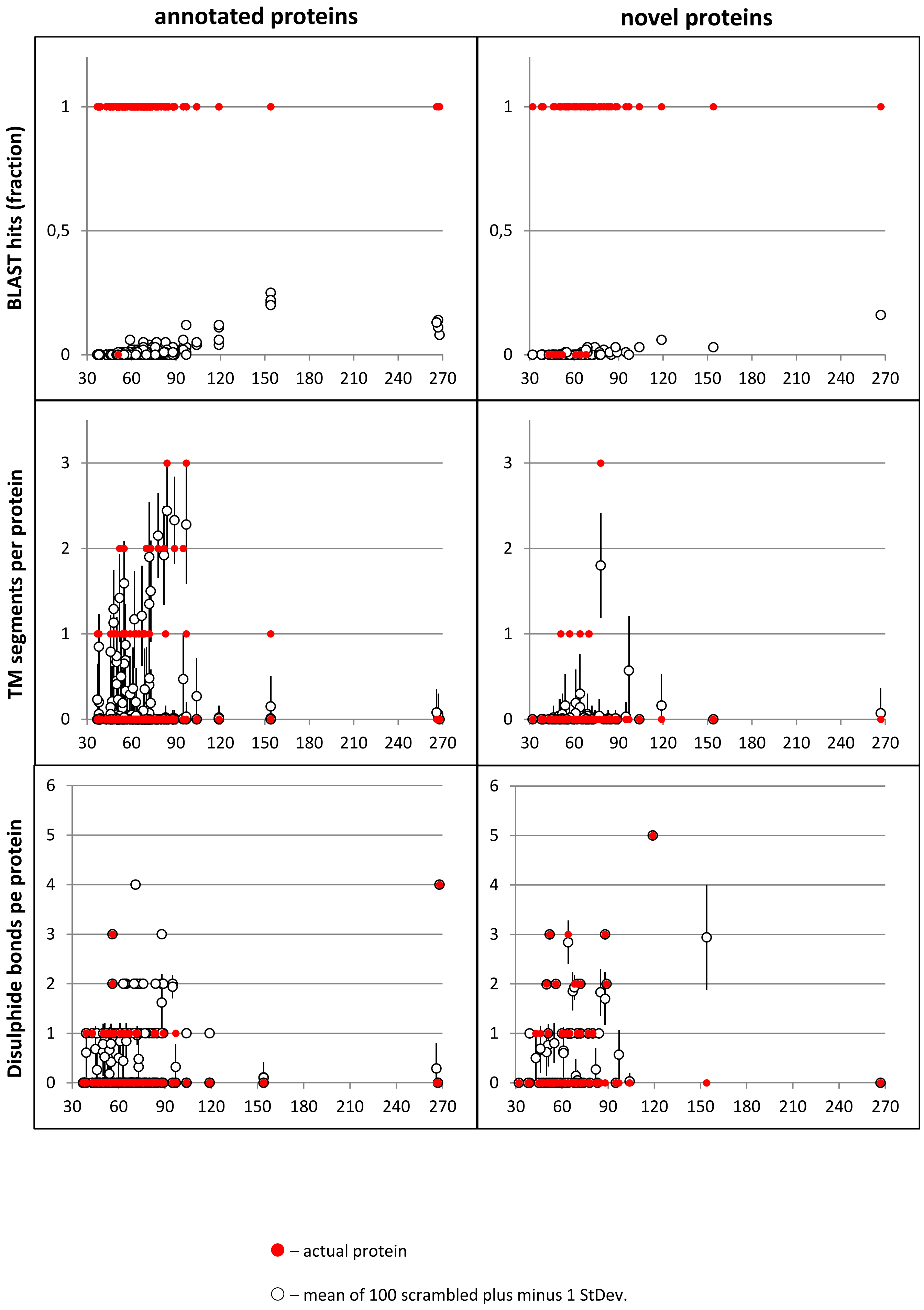
annotated proteins
novel proteins
BLAST hits (fraction)
TM segments per protein
Disulphide bonds pe protein
– actual protein
– mean of 100 scrambled plus minus 1 StDev.

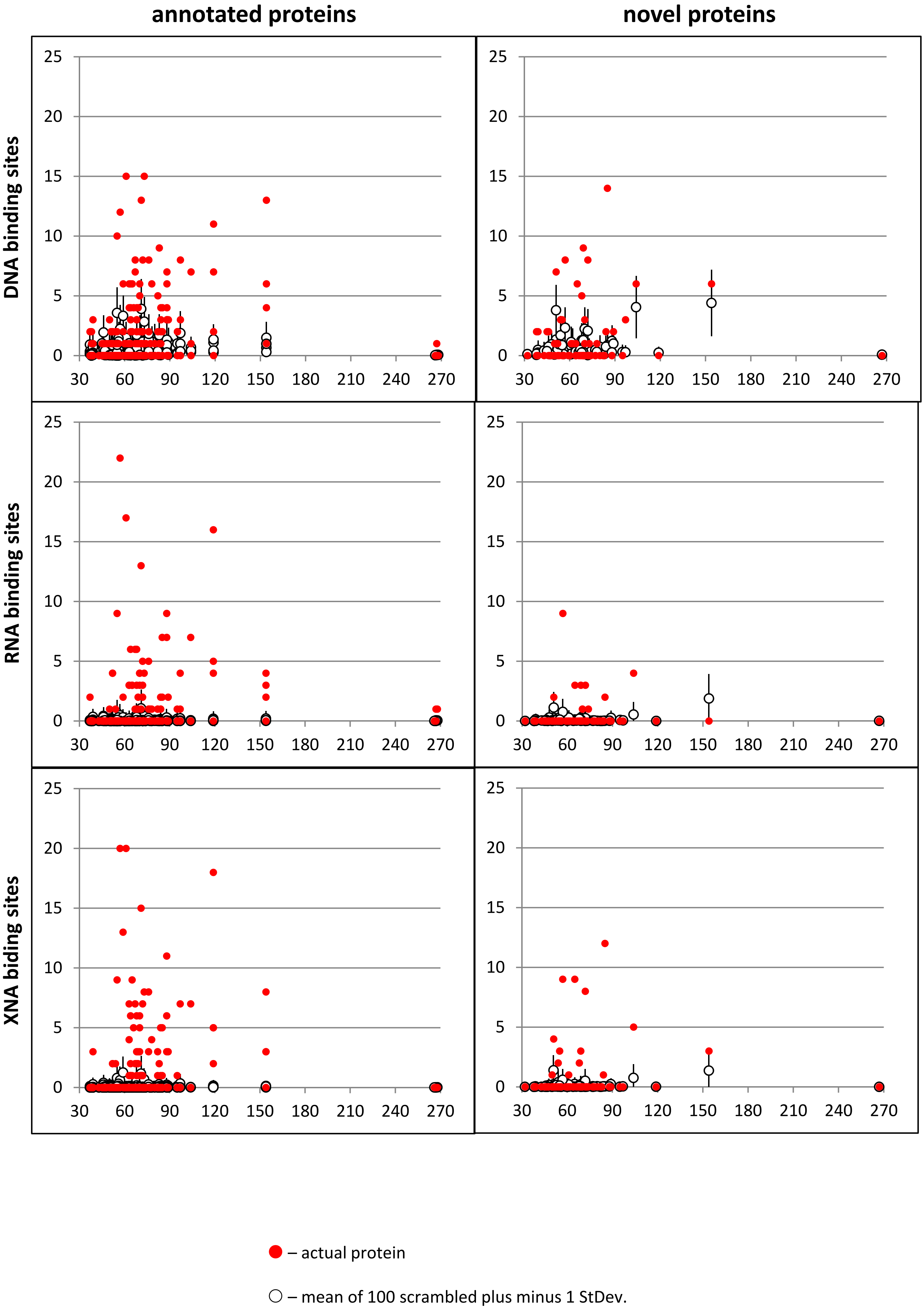
annotated proteins
novel proteins
DNA binding sites
RNA binding sites
XNA biding sites
0
5
10
15
20
25
30
60
90
120
150
180
210
240
270
– actual protein
– mean of 100 scrambled plus minus 1 StDev.

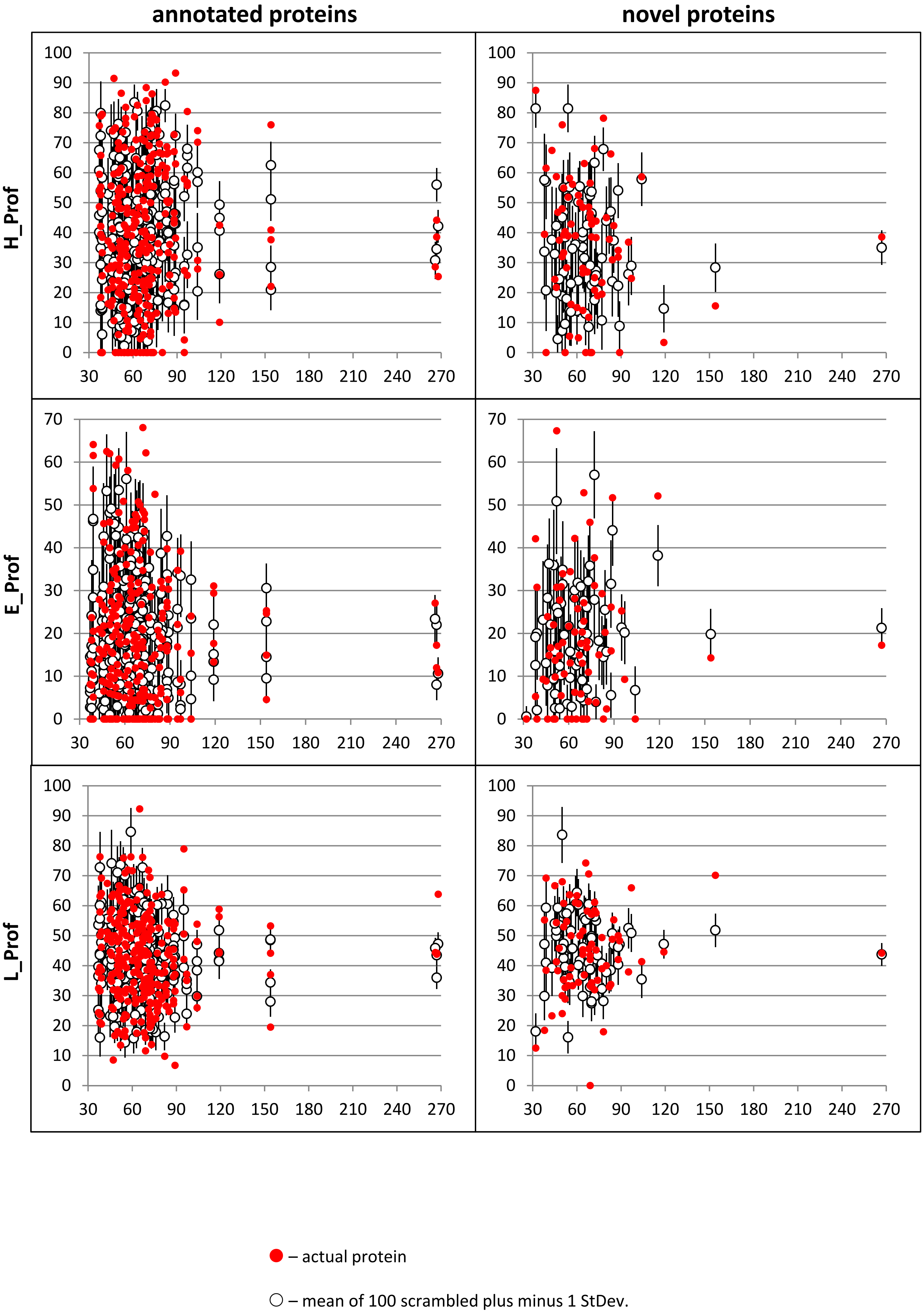
annotated proteins
novel proteins
H_Prof
E_Prof
L_Prof
– actual protein
– mean of 100 scrambled plus minus 1 StDev.

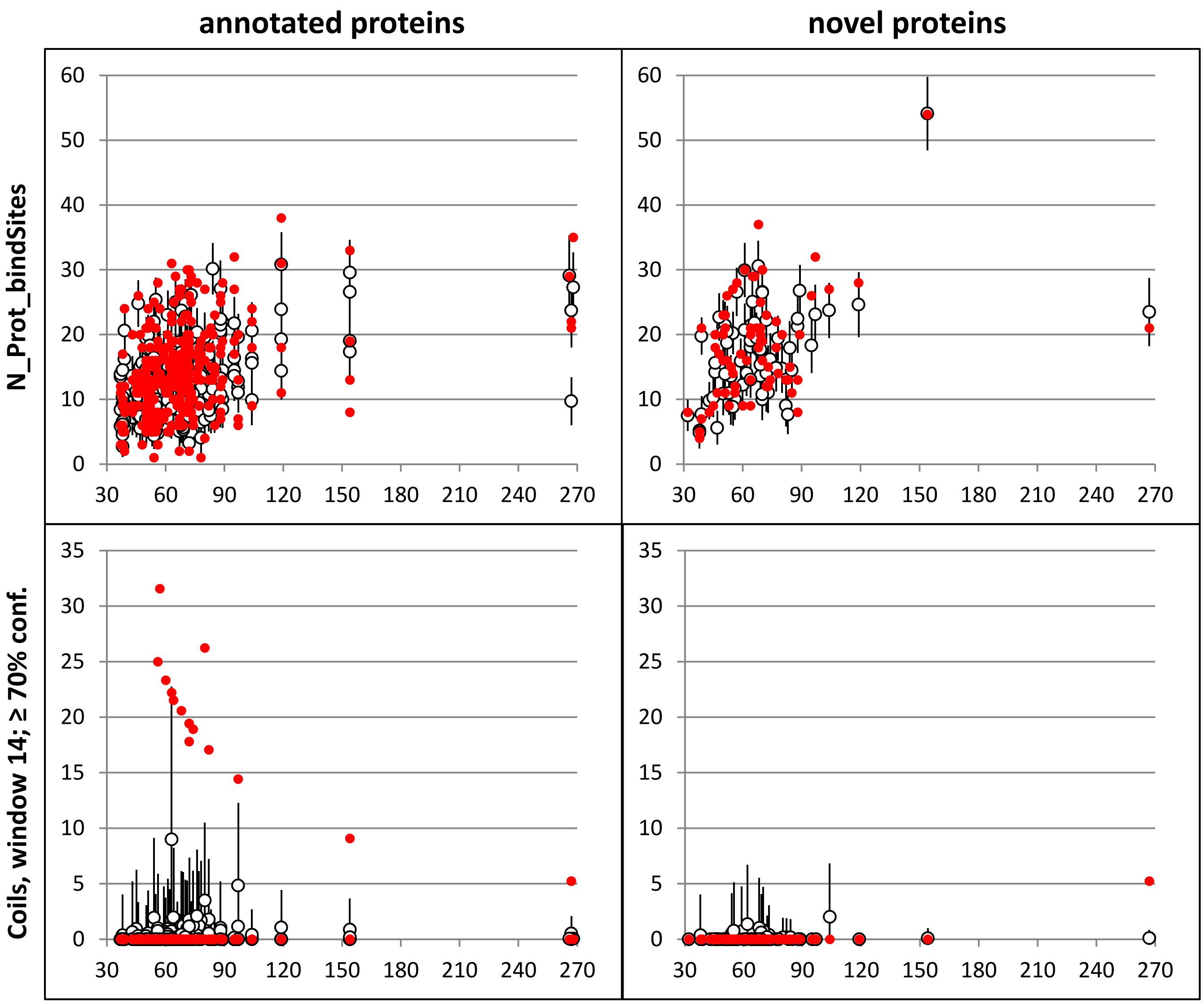

● – actual protein

○ – mean of 100 scrambled plus minus 1 StDev.

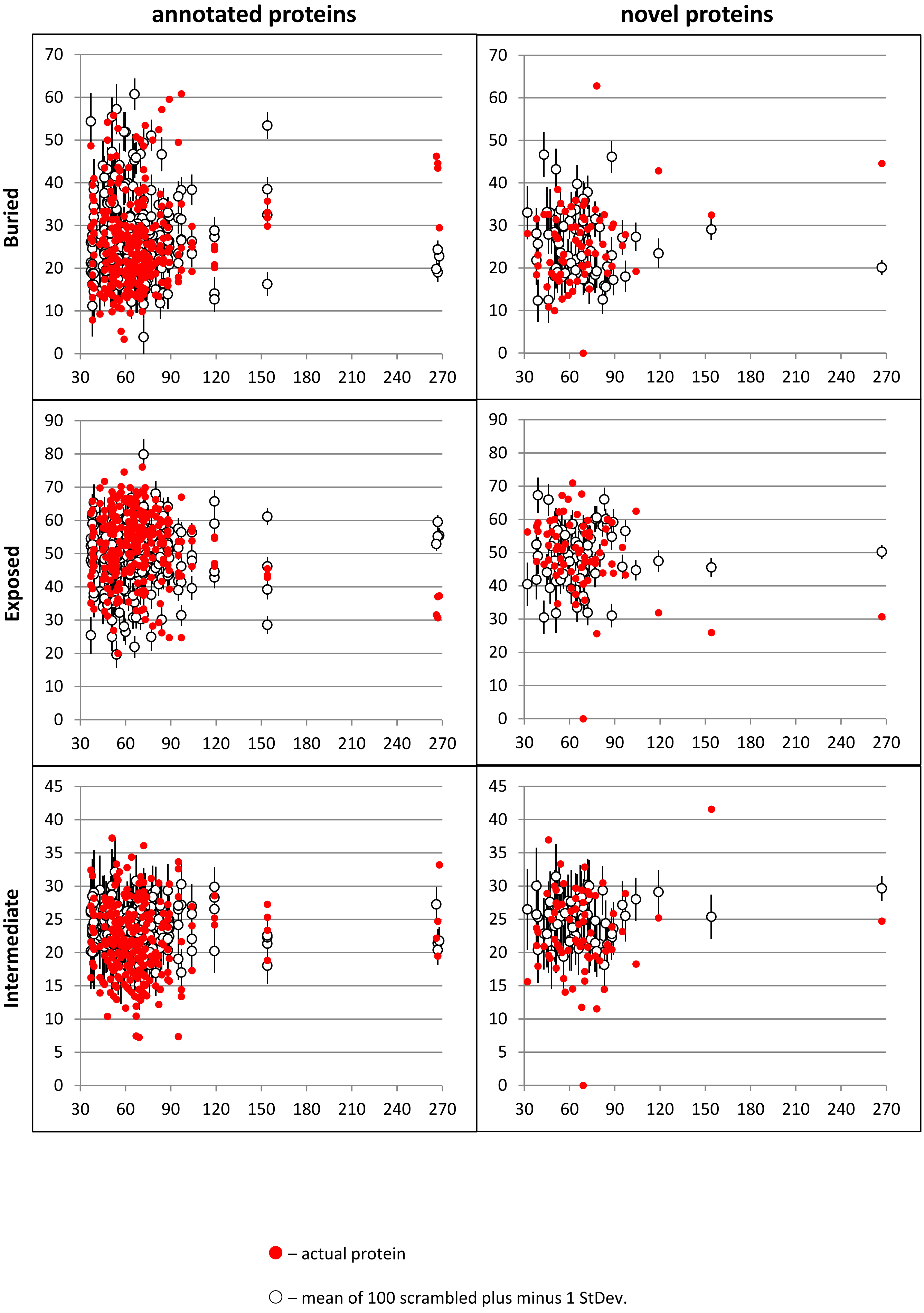
annotated proteins
novel proteins
Buried
Exposed
Intermediate
– actual protein
– mean of 100 scrambled plus minus 1 StDev.

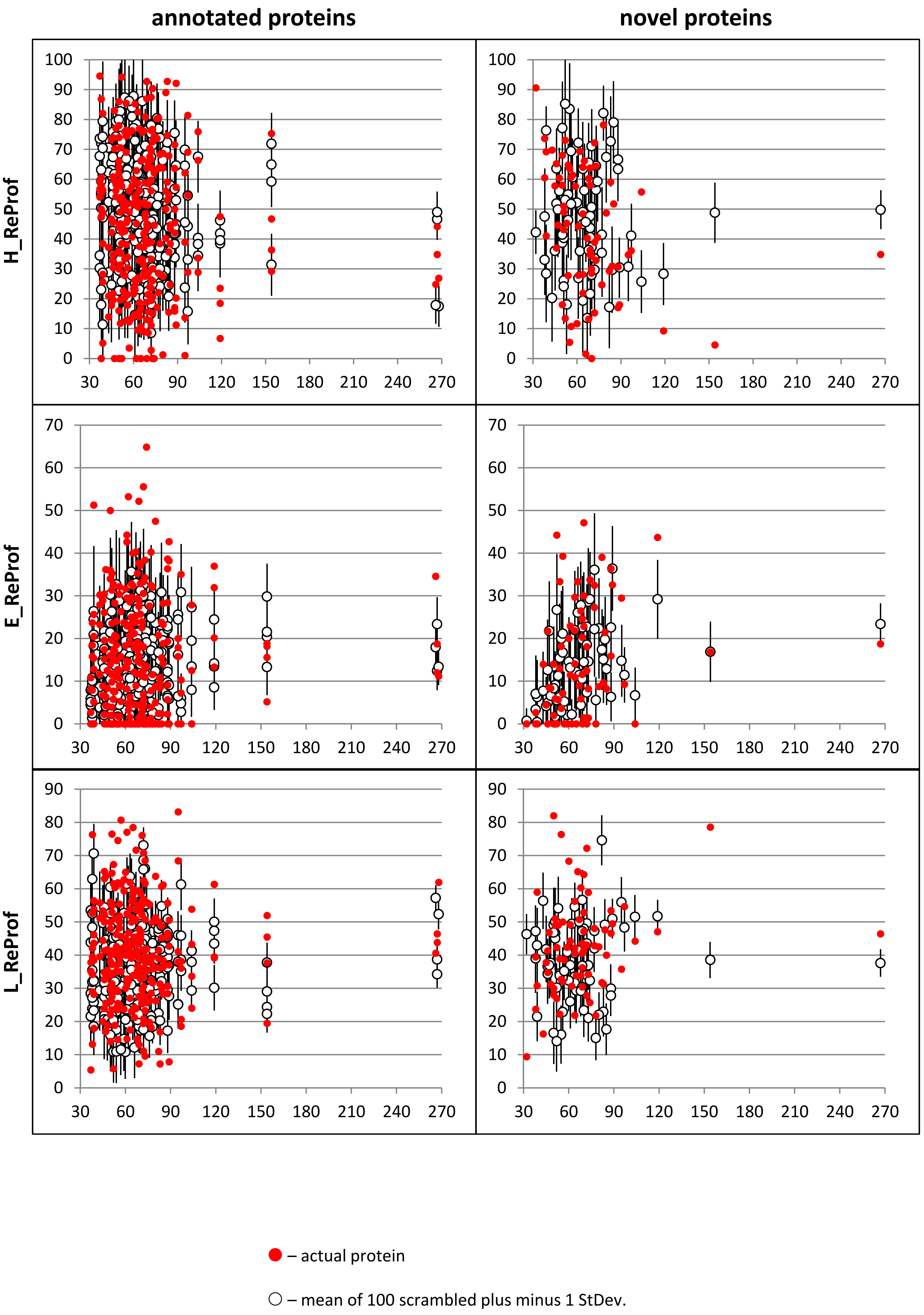

annotated proteins
novel proteins
H_ReProf
E_ReProf
L_ReProf
– actual protein
– mean of 100 scrambled plus minus 1 StDev.

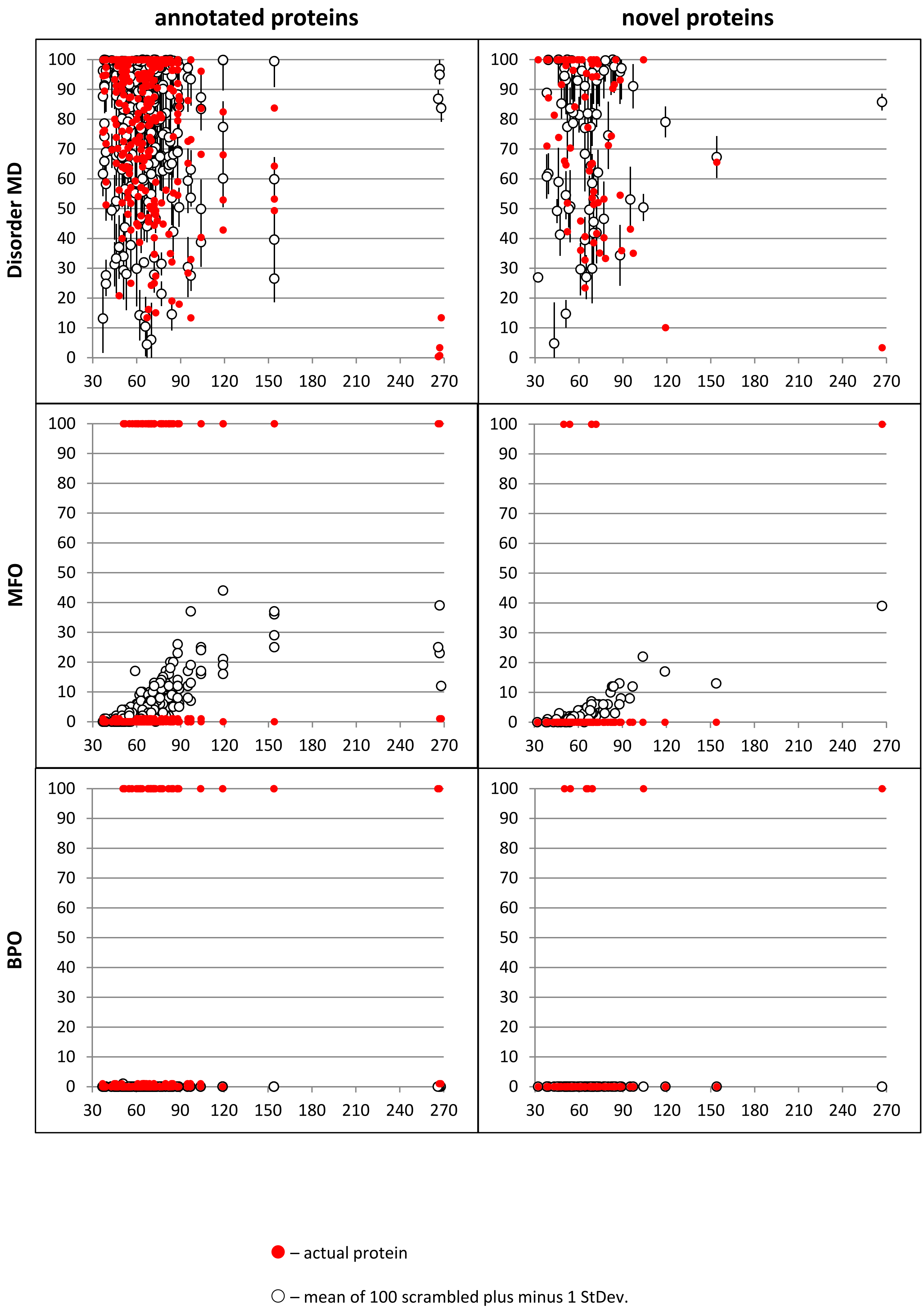
annotated proteins
novel proteins
Disorder MD
MFO
BPO
100
90
80
70
60
50
40
30
20
10
0
30
60
90
120
150
180
210
240
270
– actual protein
– mean of 100 scrambled plus minus 1 StDev.

cellular localization

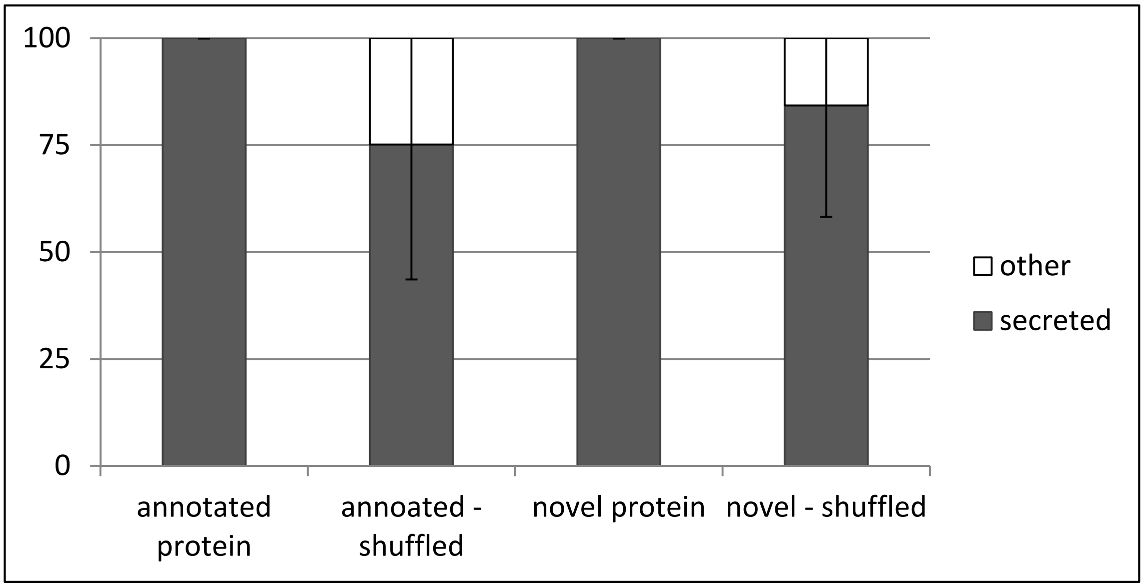

amino acid composition

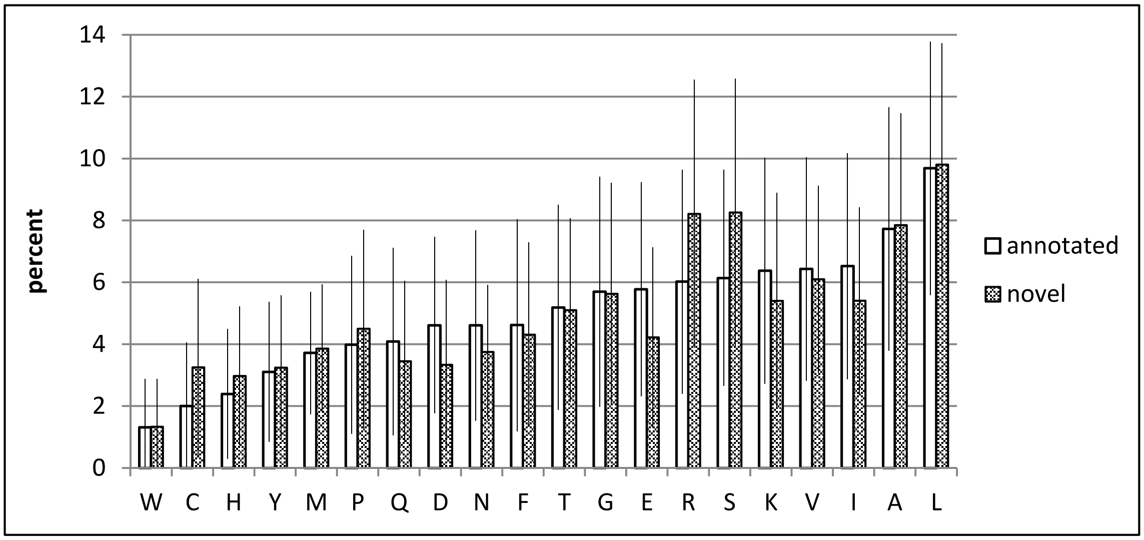

Supplement: Additional file 5: — Prediction values for the novel and annotated proteins compared to their shuffled counterparts in dependence of the protein length. (PDF 443 kb) [file 12864_2016_2456_MOESM5_ESM.pdf]
