# Supplementary material for: Translatomics combined with transcriptomics and proteomics reveals novel functional, recently evolved orphan genes in Escherichia coli O157:H7 (EHEC)
Source: BMC Genomics. 2016 Feb 24;17:133. doi: 10.1186/s12864-016-2456-1 (PMC4765031; doi:10.1186/s12864-016-2456-1)
Supplement: Additional file 6: — Prediction values for the novel proteins compared to their shuffled counterparts for each protein individually. (PDF 144 kb) [file 12864_2016_2456_MOESM6_ESM.pdf]

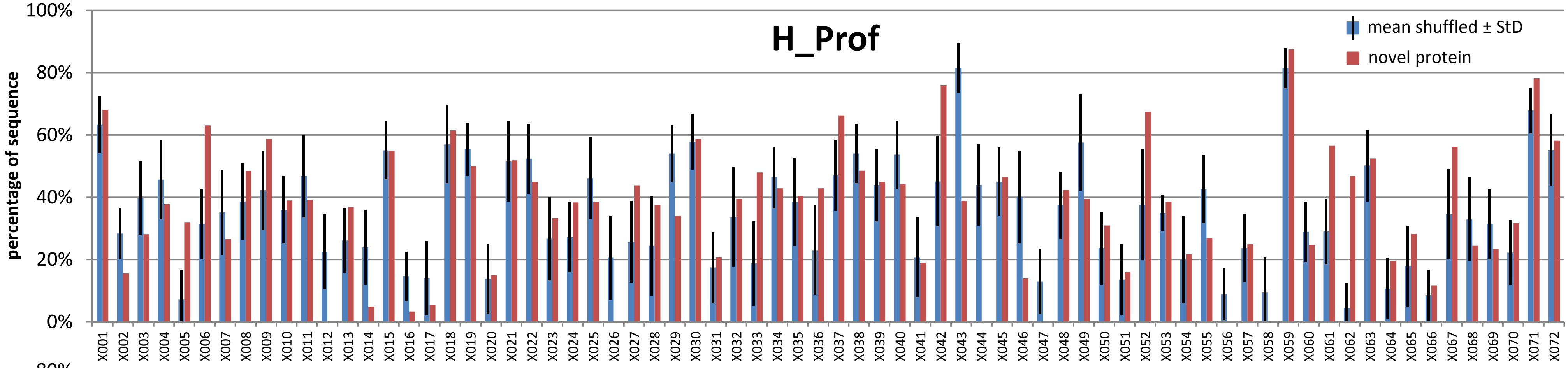
H_Prof
mean shuffled ± StD
novel protein
percentage of sequence
100%
80%
60%
40%
20%
0%

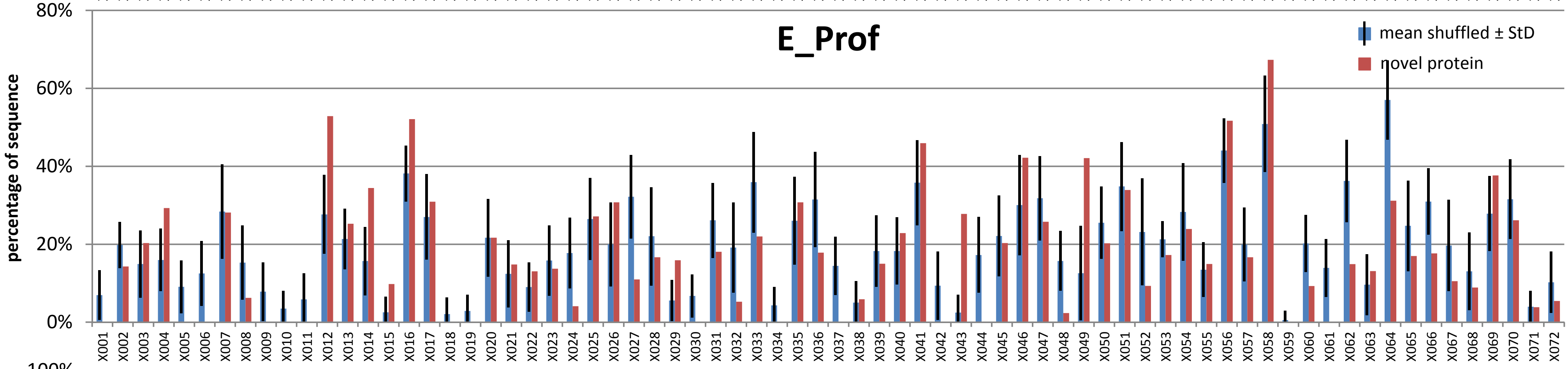
E_Prof
mean shuffled ± StD
novel protein
percentage of sequence
80%
60%
40%
20%
0%

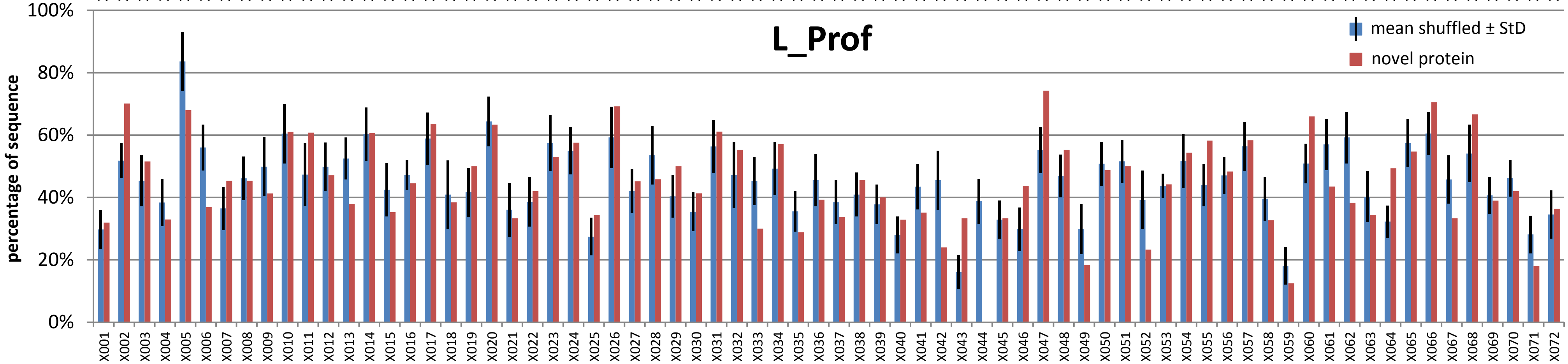
L_Prof
mean shuffled ± StD
novel protein
percentage of sequence
100%
80%
60%
40%
20%
0%

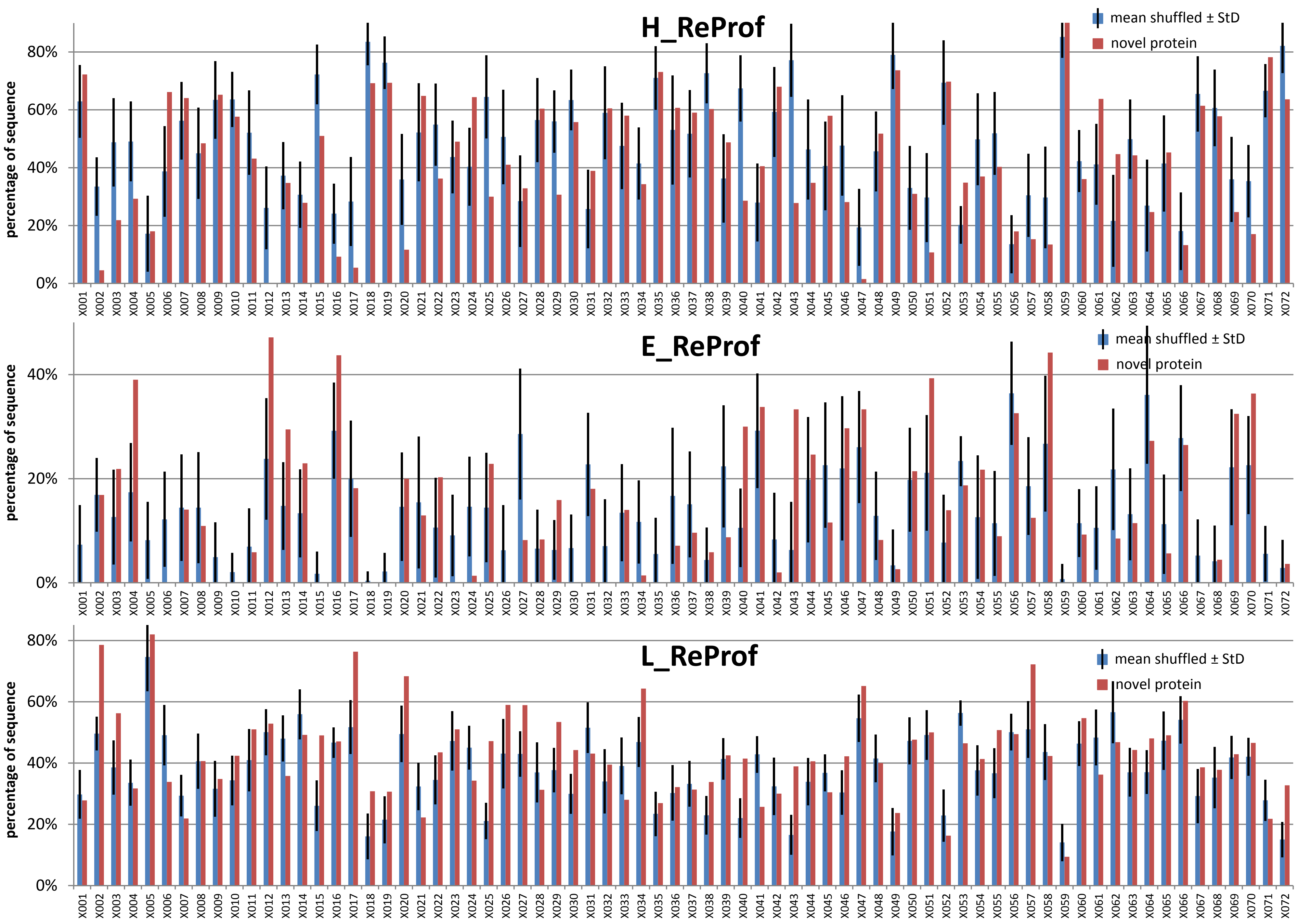
H_ReProf
mean shuffled ± StD
novel protein
percentage of sequence
0%
20%
40%
60%
80%
E_ReProf
0%
20%
40%
L_ReProf

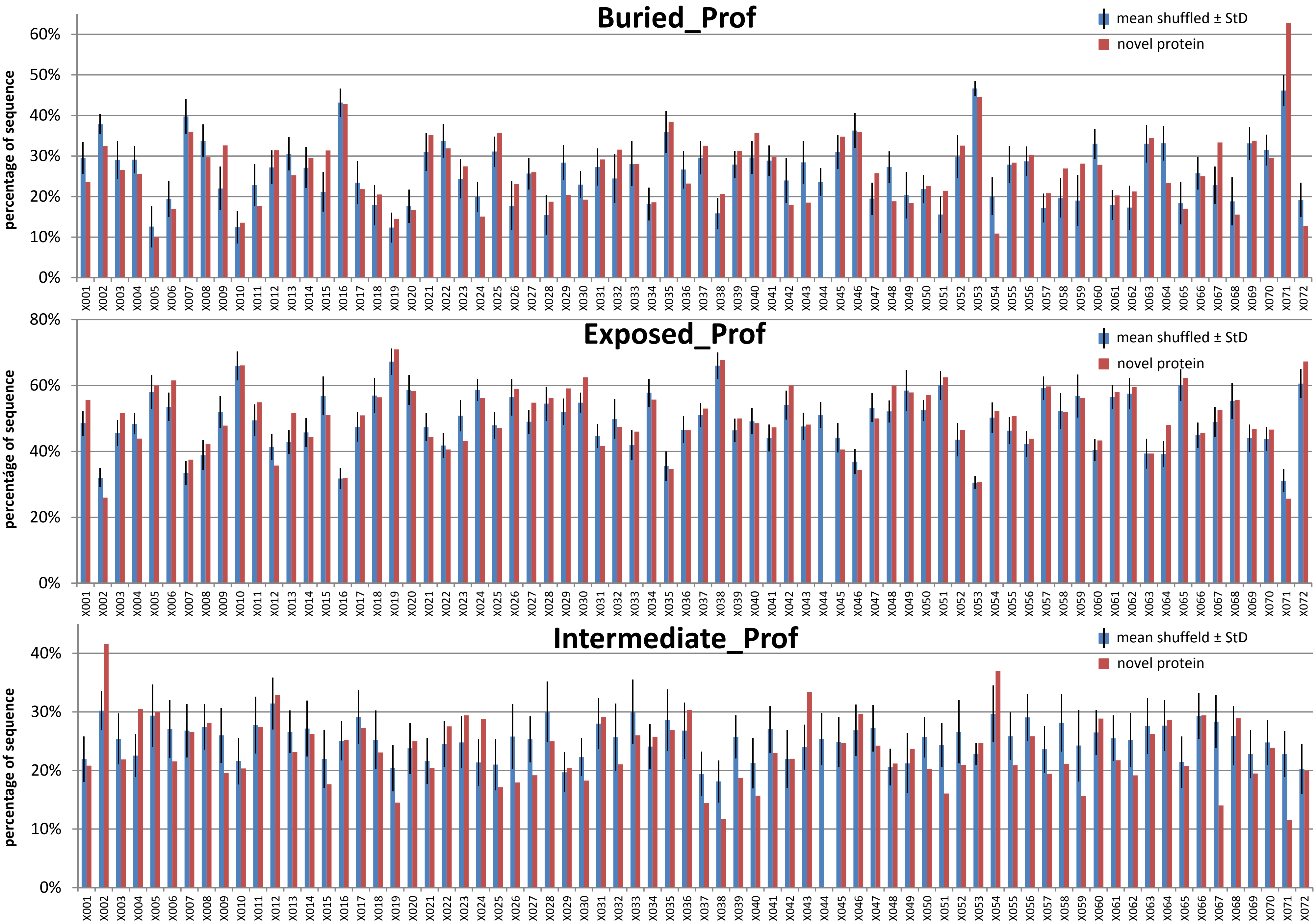
Buried_Prof
mean shuffled ± StD
novel protein
percentage of sequence
0% 10% 20% 30% 40% 50% 60%
Exposed_Prof
mean shuffled ± StD
novel protein
percentage of sequence
0% 20% 40% 60% 80%
Intermediate_Prof
mean shuffeld ± StD
novel protein
percentage of sequence
0% 10% 20% 30% 40%
X001 X002 X003 X004 X005 X006 X007 X008 X009 X010 X011 X012 X013 X014 X015 X016 X017 X018 X019 X020 X021 X022 X023 X024 X025 X026 X027 X028 X029 X030 X031 X032 X033 X034 X035 X036 X037 X038 X039 X040 X041 X042 X043 X044 X045 X046 X047 X048 X049 X050 X051 X052 X053 X054 X055 X056 X057 X058 X059 X060 X061 X062 X063 X064 X065 X066 X067 X068 X069 X070 X071 X072

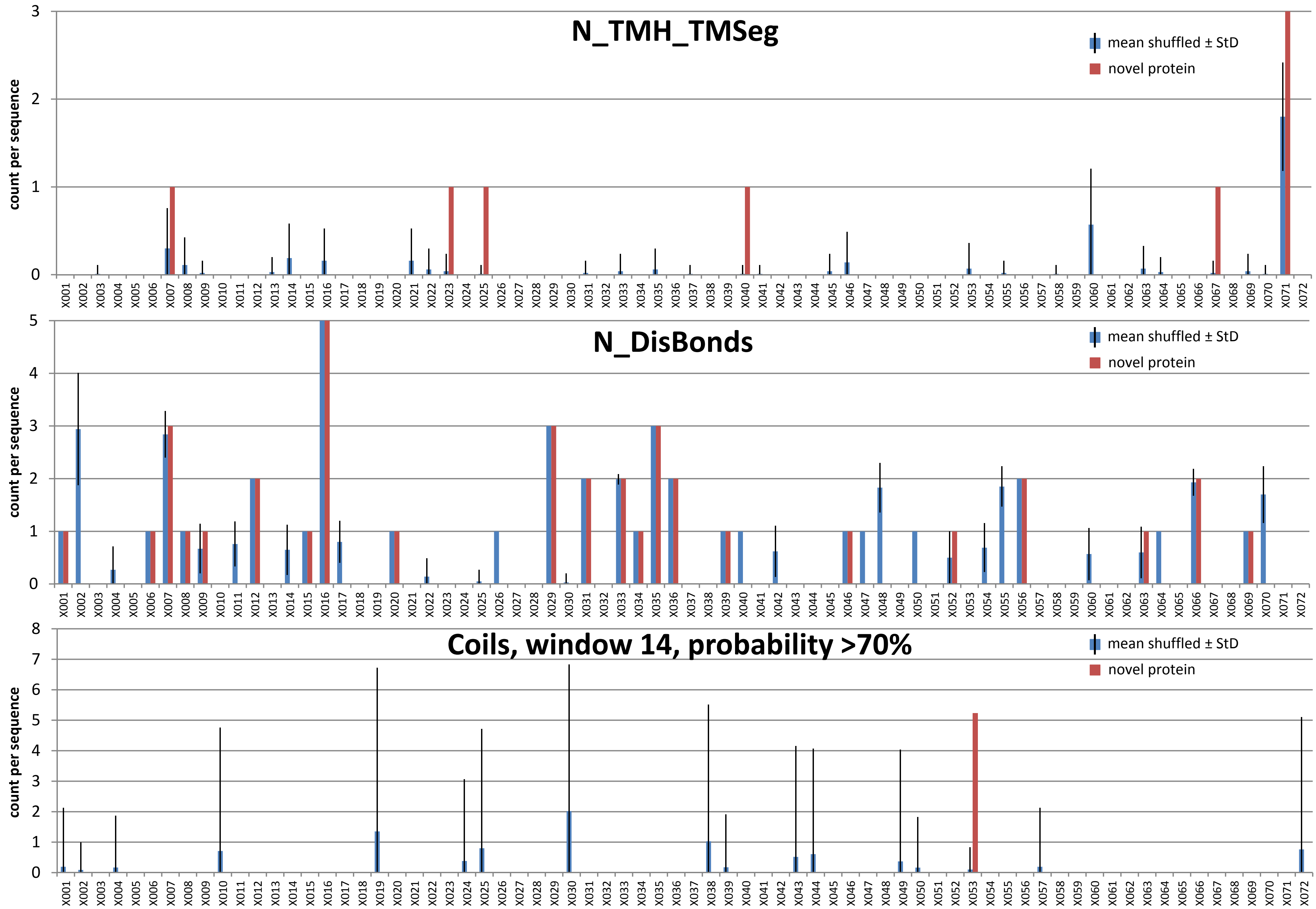

N_TMH_TMSeg
mean shuffled ± StD
novel protein
count per sequence
0 1 2 3
X001 X002 X003 X004 X005 X006 X007 X008 X009 X010 X011 X012 X013 X014 X015 X016 X017 X018 X019 X020 X021 X022 X023 X024 X025 X026 X027 X028 X029 X030 X031 X032 X033 X034 X035 X036 X037 X038 X039 X040 X041 X042 X043 X044 X045 X046 X047 X048 X049 X050 X051 X052 X053 X054 X055 X056 X057 X058 X059 X060 X061 X062 X063 X064 X065 X066 X067 X068 X069 X070 X071 X072
N_DisBonds
mean shuffled ± StD
novel protein
count per sequence
0 1 2 3 4 5
X001 X002 X003 X004 X005 X006 X007 X008 X009 X010 X011 X012 X013 X014 X015 X016 X017 X018 X019 X020 X021 X022 X023 X024 X025 X026 X027 X028 X029 X030 X031 X032 X033 X034 X035 X036 X037 X038 X039 X040 X041 X042 X043 X044 X045 X046 X047 X048 X049 X050 X051 X052 X053 X054 X055 X056 X057 X058 X059 X060 X061 X062 X063 X064 X065 X066 X067 X068 X069 X070 X071 X072
Coils, window 14, probability >70%
mean shuffled ± StD
novel protein
count per sequence
0 1 2 3 4 5 6 7 8
X001 X002 X003 X004 X005 X006 X007 X008 X009 X010 X011 X012 X013 X014 X015 X016 X017 X018 X019 X020 X021 X022 X023 X024 X025 X026 X027 X028 X029 X030 X031 X032 X033 X034 X035 X036 X037 X038 X039 X040 X041 X042 X043 X044 X045 X046 X047 X048 X049 X050 X051 X052 X053 X054 X055 X056 X057 X058 X059 X060 X061 X062 X063 X064 X065 X066 X067 X068 X069 X070 X071 X072

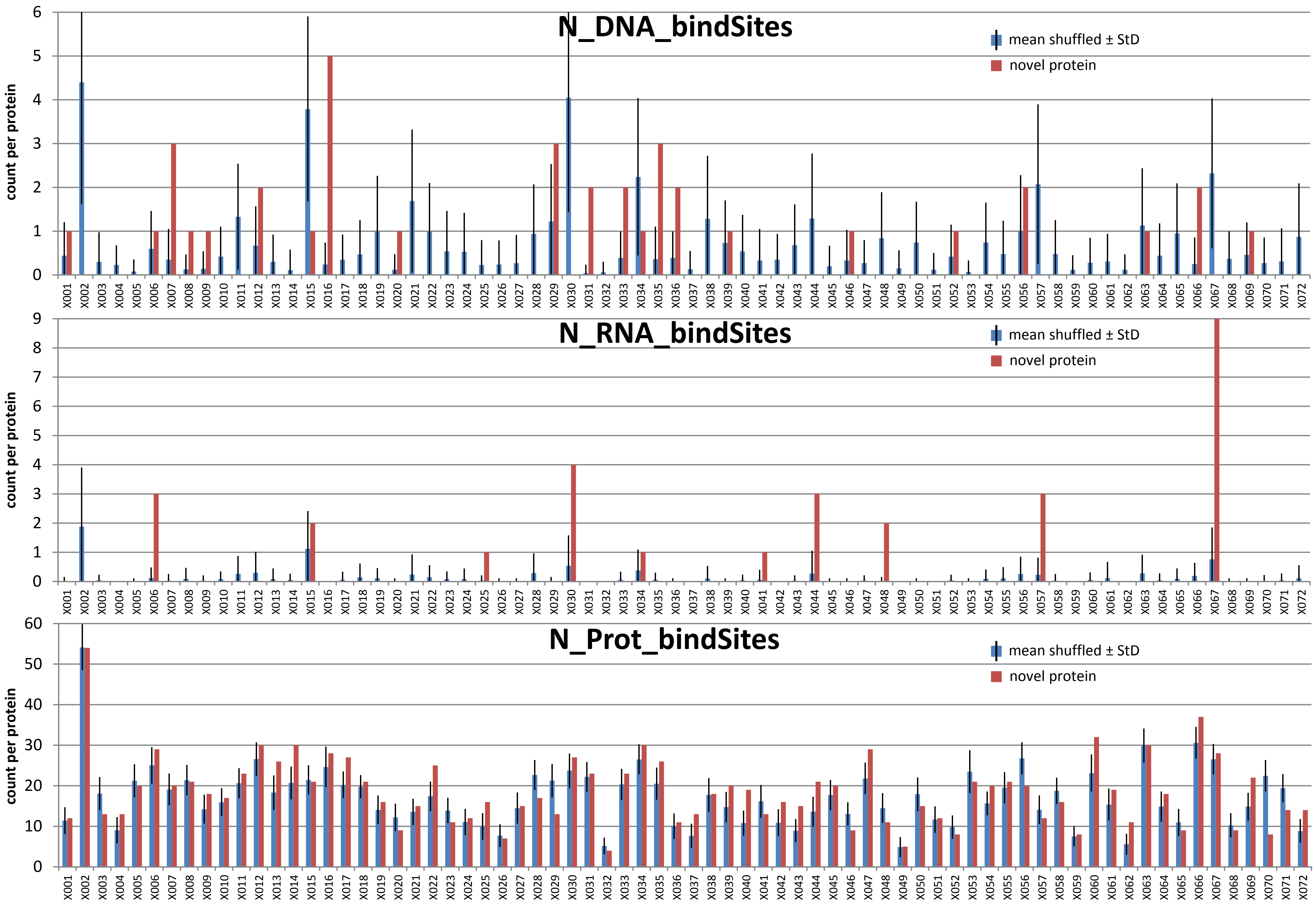
N_DNA_bindSites
mean shuffled ± StD
novel protein
count per protein
N_RNA_bindSites
mean shuffled ± StD
novel protein
count per protein
N_Prot_bindSites
mean shuffled ± StD
novel protein
count per protein
X001 … X072

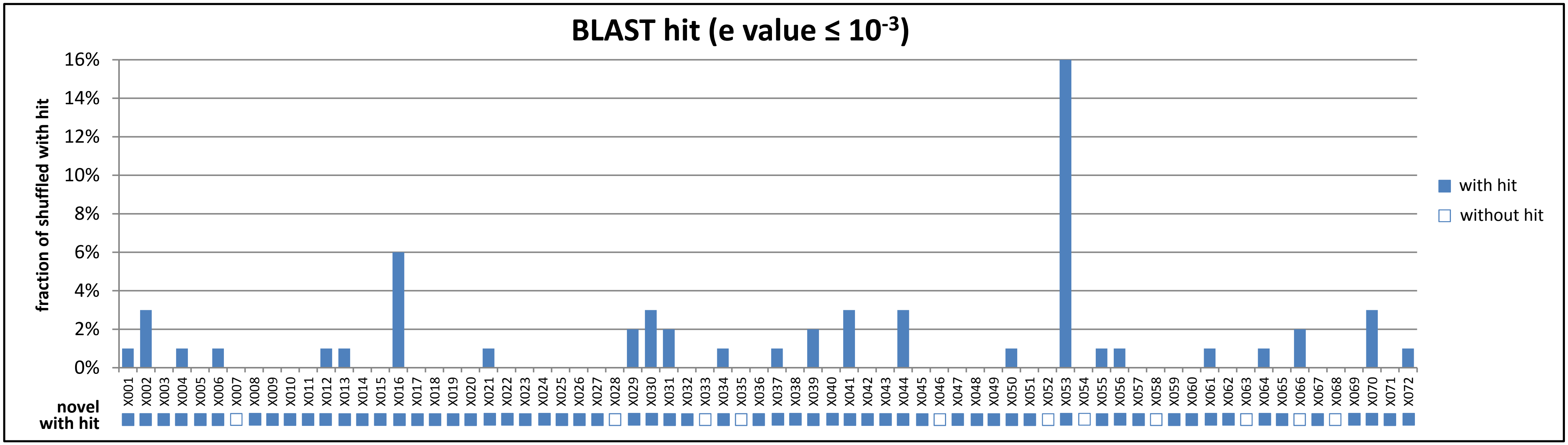
BLAST hit (e value ≤ 10-3)
fraction of shuffled with hit
16%
14%
12%
10%
8%
6%
4%
2%
0%
with hit
without hit
novel with hit

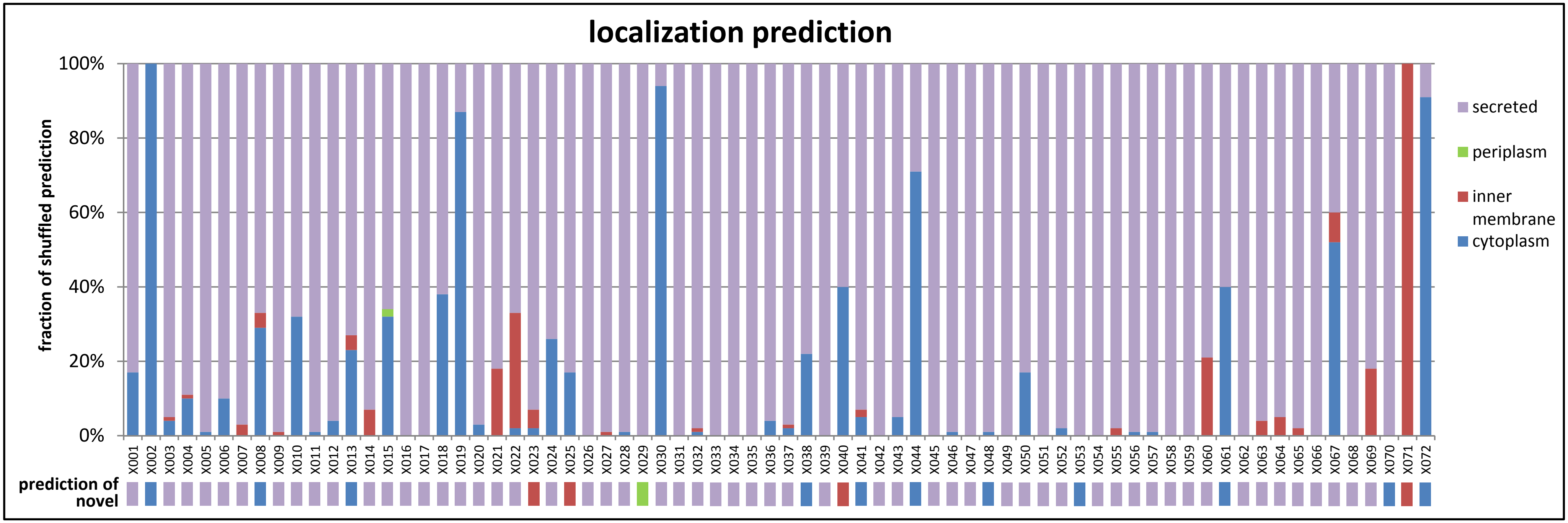
localization prediction
fraction of shuffled prediction
100%
80%
60%
40%
20%
0%
secreted
periplasm
inner membrane
cytoplasm
prediction of novel

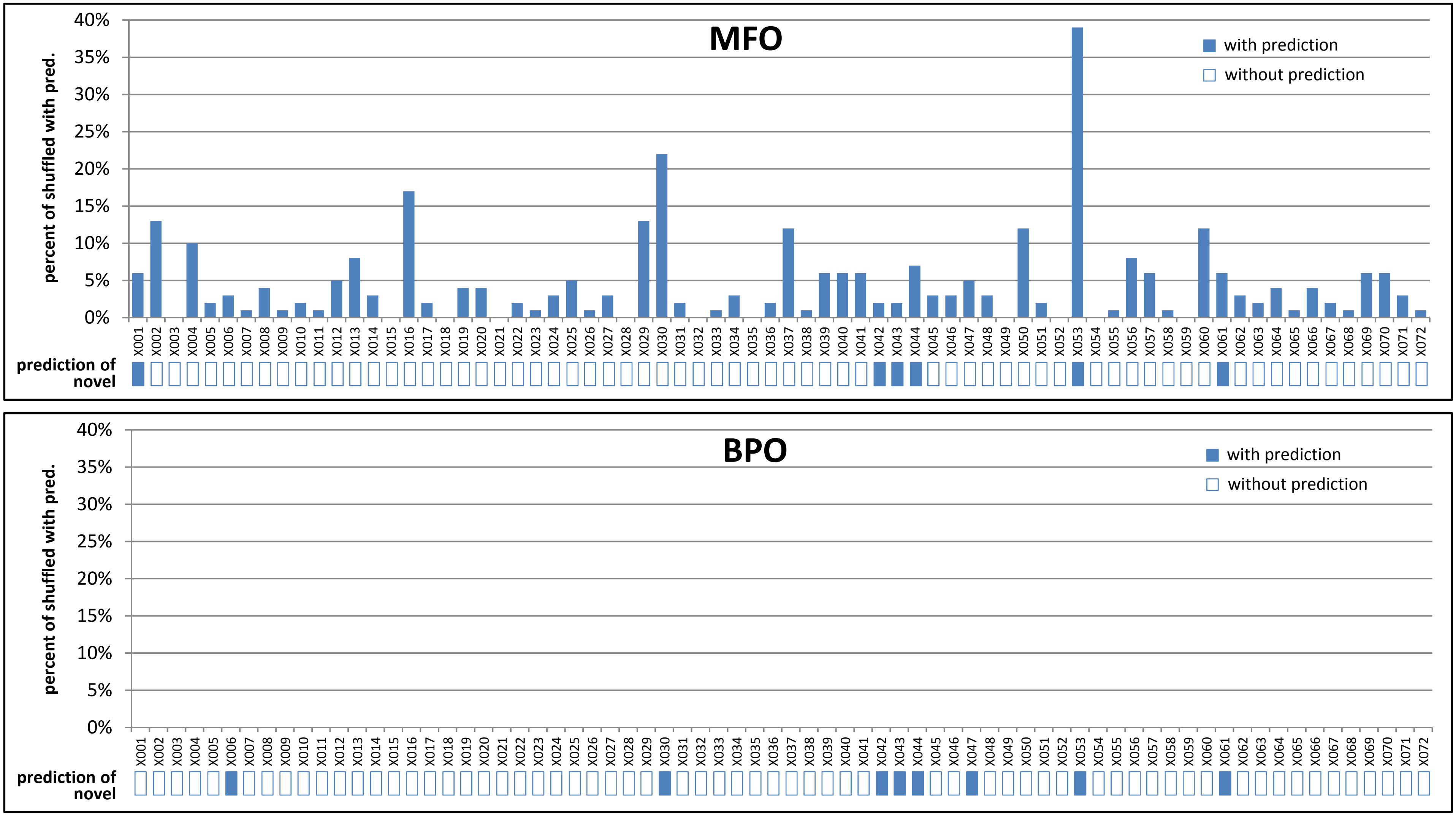
MFO
BPO
percent of shuffled with pred.
40%
35%
30%
25%
20%
15%
10%
5%
0%
with prediction
without prediction
prediction of novel
X001 … X072

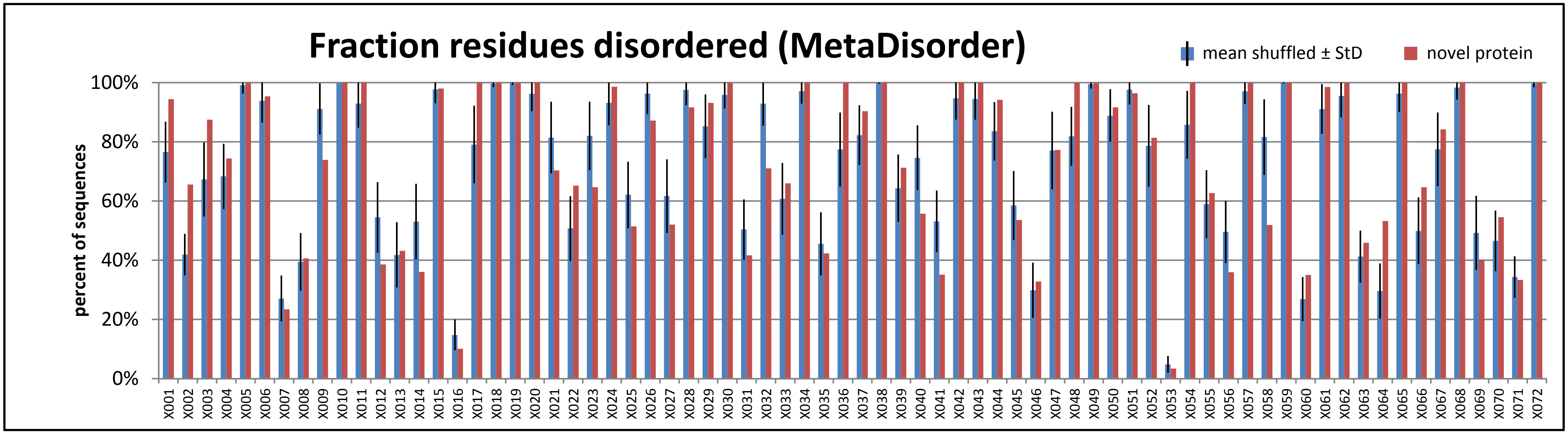

Fraction residues disordered (MetaDisorder)
mean shuffled ± StD
novel protein
percent of sequences
100%
80%
60%
40%
20%
0%
X001 X002 X003 X004 X005 X006 X007 X008 X009 X010 X011 X012 X013 X014 X015 X016 X017 X018 X019 X020 X021 X022 X023 X024 X025 X026 X027 X028 X029 X030 X031 X032 X033 X034 X035 X036 X037 X038 X039 X040 X041 X042 X043 X044 X045 X046 X047 X048 X049 X050 X051 X052 X053 X054 X055 X056 X057 X058 X059 X060 X061 X062 X063 X064 X065 X066 X067 X068 X069 X070 X071 X072

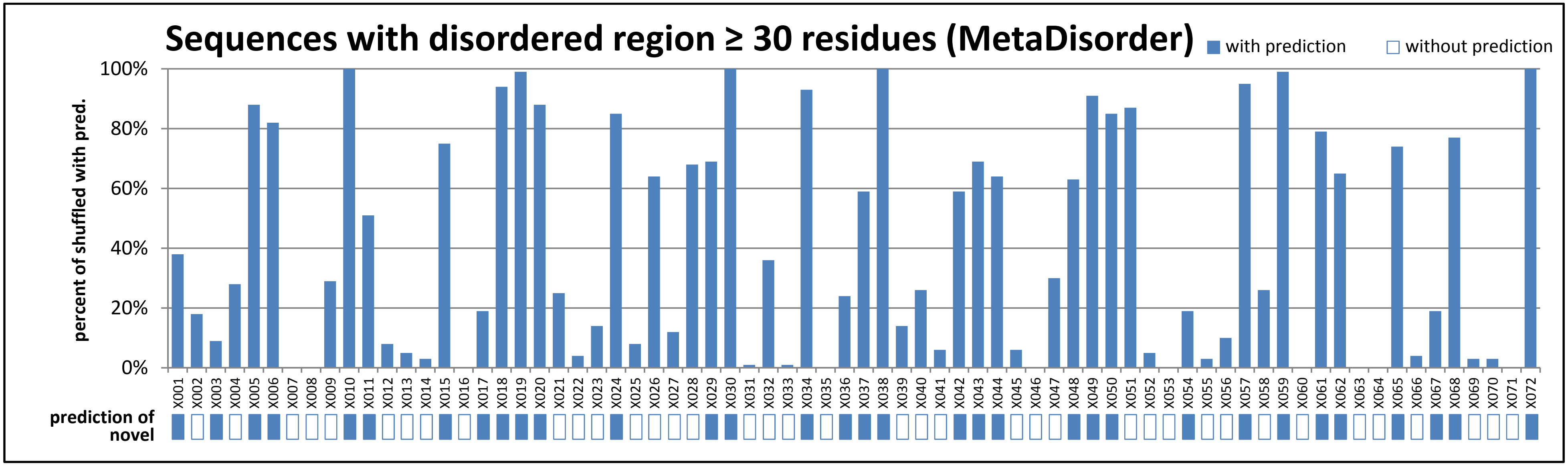

Sequences with disordered region ≥ 30 residues (MetaDisorder)
with prediction
without prediction
percent of shuffled with pred.
100%
80%
60%
40%
20%
0%
X001 X002 X003 X004 X005 X006 X007 X008 X009 X010 X011 X012 X013 X014 X015 X016 X017 X018 X019 X020 X021 X022 X023 X024 X025 X026 X027 X028 X029 X030 X031 X032 X033 X034 X035 X036 X037 X038 X039 X040 X041 X042 X043 X044 X045 X046 X047 X048 X049 X050 X051 X052 X053 X054 X055 X056 X057 X058 X059 X060 X061 X062 X063 X064 X065 X066 X067 X068 X069 X070 X071 X072
prediction of novel
