# Supplementary material for: Translatomics combined with transcriptomics and proteomics reveals novel functional, recently evolved orphan genes in Escherichia coli O157:H7 (EHEC)
Source: BMC Genomics. 2016 Feb 24;17:133. doi: 10.1186/s12864-016-2456-1 (PMC4765031; doi:10.1186/s12864-016-2456-1)
Supplement: Additional file 9: — Cumulative distributions of PSI-Blast and HHblits family sizes. (PDF 8541 kb) [file 12864_2016_2456_MOESM9_ESM.pdf]

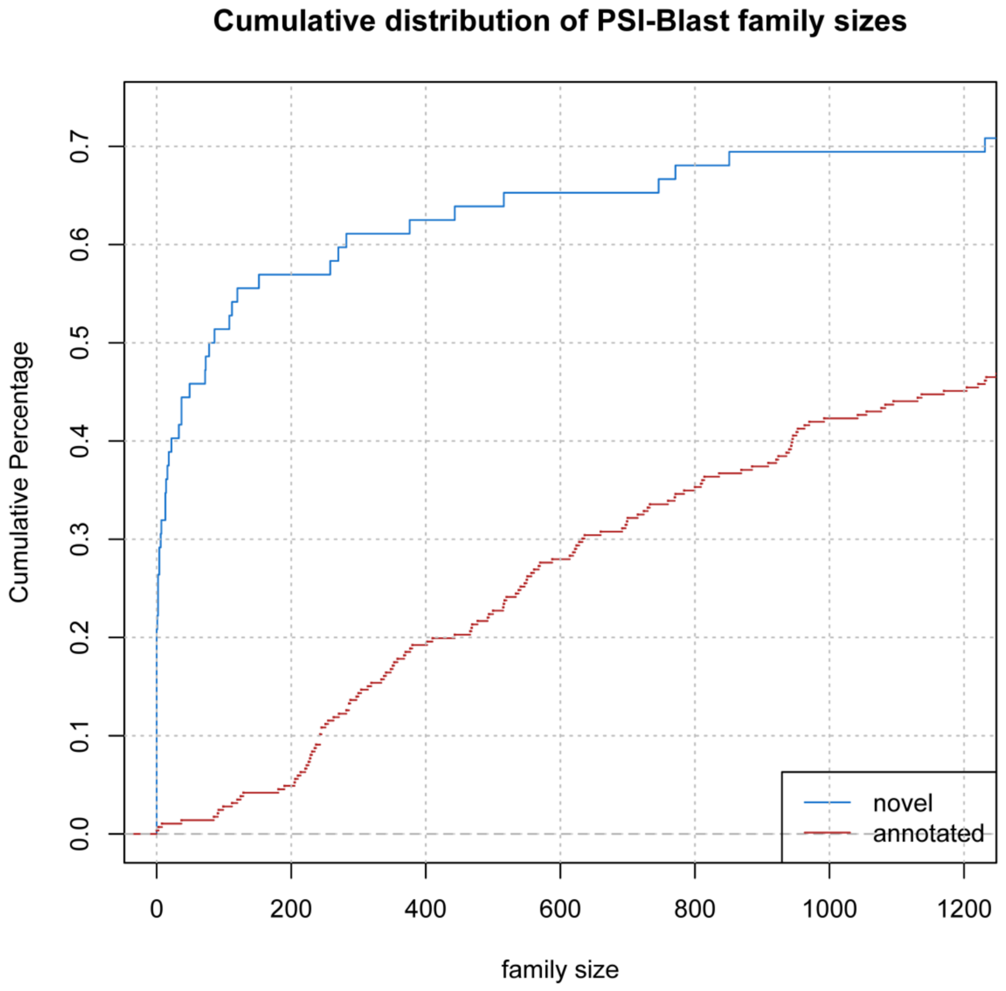

PSI-Blast search at an E value of ≤10^{-3}. At this E-Value cut-off we count for each query how many subjects we receive in Uniprot, which is defined as „family size“. Shown is on the y-axis the cumulative percentage of the proteins (novel, blue; annotated, red) which have a family size of the same size or smaller than this given percentage of the proteins. E.g., at a family size of 200, about 57% of the novel proteins have a family size of 200 or less, but only about 5% of the annotated proteins have a family size of 200 or less. More important than the actual values is the trend, since the E-value or any chosen percentage / family size combination are of arbitrary choice. However, most of the novel proteins have small family sizes compared to the annotated.

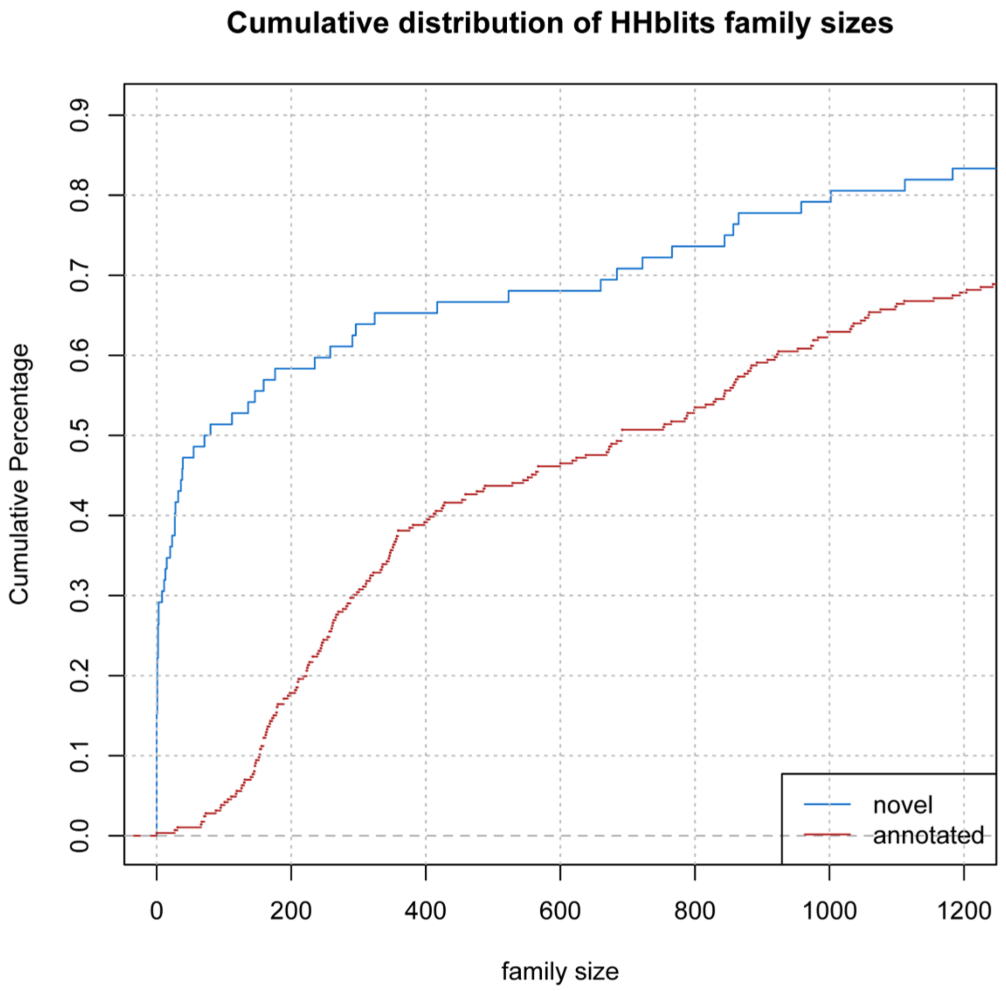

HHblits search at an E value of ≤10^{-3}. At this E-Value cut-off we count for each query how many subjects we receive in Uniprot, which is defined as „family size". Shown is on the y-axis the cumulative percentage of the proteins (novel, blue; annotated, red) which have a family size of the same size or smaller than this given percentage of the proteins. E.g., at a family size of 200, about 59% of the novel proteins have a family size of 200 or less, but only about 18% of the annotated proteins have a family size of 200 or less. More important than the actual values is the trend, since the E-value or any chosen percentage / family size combination are of arbitrary choice. However, most of the novel proteins have small family sizes compared to the annotated.
